# Iron Biofortification and Homeostasis in Transgenic Cassava Roots Expressing the Algal Iron Assimilatory Gene, *FEA1*

**DOI:** 10.3389/fpls.2012.00171

**Published:** 2012-09-13

**Authors:** Uzoma E. Ihemere, Narayanan N. Narayanan, Richard T. Sayre

**Affiliations:** ^1^Donald Danforth Plant Science CenterSt. Louis, MO, USA

**Keywords:** iron, FEA1, cassava, biofortification, *Chlamydomonas*, malnutrition

## Abstract

We have engineered the tropical root crop cassava (*Manihot esculenta*) to express the *Chlamydomonas reinhardtii* iron assimilatory gene, *FEA1*, in its storage roots with the objective of enhancing the root nutritional qualities. Iron levels in mature cassava storage roots were increased from 10 to 36 ppm in the highest iron accumulating transgenic lines. These iron levels are sufficient to meet the minimum daily requirement for iron in a 500 g meal. Significantly, the expression of the *FEA1* gene in storage roots did not alter iron levels in leaves. Transgenic plants also had normal levels of zinc in leaves and roots consistent with the specific uptake of ferrous iron mediated by the FEA1 protein. Relative to wild-type plants, fibrous roots of *FEA1* expressing plants had reduced Fe (III) chelate reductase activity consistent with the more efficient uptake of iron in the transgenic plants. We also show that multiple cassava genes involved in iron homeostasis have altered tissue-specific patterns of expression in leaves, stems, and roots of transgenic plants consistent with increased iron sink strength in transgenic roots. These results are discussed in terms of strategies for the iron biofortification of plants.

## Introduction

Iron deficiency anemia impacts over 50% of the world’s population and if untreated can cause reduced psychomotor and mental development in children, aborted pregnancies, and compromised immunity (Murphy et al., 1986; Murakawa et al., 1987; Savoie and Rioux, 2002; Nestel et al., 2006; Zimmermann and Hurrell, 2007). Significantly, nearly 90% of the global population impacted by iron-deficient anemia lives in Southeast Asia and Africa and often subsists on only one major staple food crop (Stephenson et al., 2000).

At least four different strategies have been employed to prevent the occurrence of iron-deficient anemia in humans. These strategies include: diet diversification to include more iron-rich foods, iron supplementation by tablet, iron fortification of processed foods, and iron biofortification of staple crops that provide the majority of the calories in the diet. Each of these strategies has specific attributes and liabilities in developing countries. Diet diversification is the ideal strategy to prevent iron-deficient anemia in developed countries but may be constrained by the limited availability and high cost of diverse iron-rich food sources. Consumption of iron-containing supplements is also an effective strategy for addressing iron nutrition in developed countries, but in developing the distribution of supplements can be challenging in remote areas due to poor transportation infrastructure, and can be logistically difficult due to language and cultural barriers. Furthermore, since iron is water soluble, iron supplements must be taken more frequently than supplements for fat soluble vitamins which are more effectively stored in the body. Food fortification during commercial processing is an alternative strategy. Mineral and vitamin biofortified foods, if available, can facilitate delivery of micronutrients on a daily basis. However, access to and delivery of fortified foods also requires appropriate infrastructure and transportation systems often lacking in developing countries. Biofortification of staple crops through breeding or transgenic approaches has the potential to be a one-time investment for food fortification if the crops are adopted, accepted, and the minerals or vitamins are sufficient and bioavailable. Genetic improvement of crop or food nutrient content by breeding can be successful if there is sufficient genetic variation for the trait (Bouis, 2003). However, for some crops, there may be insufficient genetic variation to reach target nutrient levels via traditional breeding approaches (Mayer et al., 2008). Recent studies indicate that for the staple crop cassava there is insufficient genetic variation to achieve iron biofortification target levels using traditional breeding approaches (Chavez et al., 2000). When breeding approaches for enhanced nutrient content are limiting the use of recombinant DNA strategies to engineer enhanced micronutrient accumulation in plants may be required to insure adequate nutritional balance in staple crops.

While iron is very abundant in the earth’s crust (5%), the ferric form of iron is very insoluble, particularly in calcareous or high pH soils (White and Broadley, 2005). To facilitate iron uptake, plants have evolved various mechanisms to solubilize and transport iron into root hairs and throughout the plant. All plants except graminaceous plants (Strategy I) typically increase iron solubility by reducing the local pH around root hairs. This is achieved by actively pumping protons via a proton-transducing ATPase. Reduction of ferric iron to ferrous iron via ferric chelate reductase also substantially increases iron solubility. Ferrous iron is then transported into root hairs via various iron transporters (Jeong and Guerinot, 2009; Palmer and Guerinot, 2009). In graminaceous plants (Strategy II), iron uptake is facilitated by secretion of phytosiderophores that complex iron and are then transported into the plants. Some grasses, e.g., rice, use both Strategy I and Strategy II Fe uptake systems (Ishimaru et al., 2006).

To date, most of the work on iron biofortification of crops has focused on rice. Rice has been engineered to express either single genes involved in iron accumulation or a combination of genes including those encoding the iron storage protein, ferritin (Goto et al., 1999; Lucca et al., 2001, 2002; Vasconcelos et al., 2003; le Qu et al., 2005), iron chelators including the barley nicotianamine synthesis gene *HvNAS* (Masuda et al., 2008), and genes that increase iron bioavailability including phytase (*Afphytase*) which degrades phytic acid. Phytic acid strongly chelates iron potentially making it bio-unavailable so its removal may increase iron bioavailablity (Lucca et al., 2001, 2002). Significantly, co-expression of nicotianamine synthesis (*AtNAS*), ferritin (*Pvferritin*), and phytase (*Afphytase*), resulted in a sixfold increase in iron accumulation in rice endosperm to approximately 6 ppm (Wirth et al., 2009).

Cassava (*Manihot esculenta*) is an important source of calories for more than 800 million people world-wide and is the staple crop for more than 300 million persons in sub-Saharan Africa. A cassava-based diet, however, does not provide adequate sources of iron, zinc, pro-vitamin A, and vitamin E (Sautter et al., 2006; Zimmermann and Hurrell, 2007; Sayre et al., 2011). Typical iron concentrations in cassava roots range from 4 to 10 ppm requiring the consumption of between 2 and 5 kg dry weight of cassava per day to meet the required daily allowance (RDA) of 18 mg iron for an adult woman (White and Broadley, 2005). Assuming an adult may consume on the order of 0.5–1 kg dry weight a day of cassava food, a typical cassava-based diet will provide less than 25% of the iron to meet the RDA for an adult woman. Recent surveys of African populations for iron-deficient anemia indicate that 43 and 47% of the population (aged between 15 and 49 years) in Kenya and Nigeria, respectively, are iron deficient (Nguema, 2011). In addition, over 188,000 and 500,000 disability adjusted life years are lost each year due to iron deficiency in Kenya and Nigeria, respectively (Nguema, 2011). Thus, societies that rely on cassava as a staple crop can often be iron deficient. Complicating the issue of meeting the RDA for iron is the fact that in many foods iron may be rendered largely non-bioavailable due to the presence of phytate which tightly binds iron and other cationic metals (Bohn et al., 2008; Wirth et al., 2009). Cassava has been reported to have varying levels of phytate, however, analyses carried out by the BioCassava Plus Program, indicated that phytate could not be detected in cassava variety TMS 60444 (Nigel Taylor, data not presented; Marfo et al., 1990; Charles et al., 2005).

We have expressed a unique algal (*Chlamydomonas reinhardtii*), iron-specific, assimilatory gene, *FEA1* in cassava storage roots with the objective of increasing their iron content. Recently, we demonstrated that the FEA1 protein was functional in yeast and plants and specifically facilitates the uptake of ferrous iron and not other elements (Narayanan et al., 2011; Leyva-Guerrero et al., 2012). Our results with transgenic cassava indicate that cassava roots expressing the *FEA1* gene have the potential to meet the RDA for iron in a typical sized 500 g meal. Significantly, the leaves of transgenic plants had normal levels of iron, thus overexpression of the *FEA1* gene in cassava roots did not result in an aberrant phenotype. In addition, there also was no significant difference in root or leaf zinc levels in *FEA1* transgenic plants consistent with the specific uptake and accumulation of iron mediated by the FEA1 protein. Over expression of the *FEA1* gene, however, was associated with altered expression of multiple genes involved in iron homeostasis in a variety of tissues consistent with increased iron sink strength in transgenic roots. These results are discussed in terms of strategies for the iron biofortification of plants for enhanced human nutrition.

## Materials and Methods

### Plant material

The cassava cultivar TMS 60444 from the International Institute for Tropical Agriculture (IITA), Ibadan, Nigeria, was used for transformation. Cassava apical leaves were placed on MS basal medium (Murashige and Skoog, 1962) supplemented with 2% (w/v) sucrose, 8 mg/L 2,4-dichlorophenoxyacetic acid (2,4-D), 10 mg/L of 100× Gamborg’s B-5 vitamins (Gamborg et al., 1968), 50 mg/L casein hydrolysate, and 0.5 mg/L CuSO_4_; pH 5.7 for the induction of somatic embryos on a 12 h/day photoperiod at 28°C at a light intensity of 50 μmol photons/m^2^/s. Germination of somatic embryos was induced by growth on MS basal medium supplemented with 1 mg/L thiamine-HCl, 100 mg/L myo-inositol, 2% (w/v) sucrose, 0.01 mg/L 2,4-D, 1.0 mg/L 6-benzylaminopurine (BAP), and 0.5 mg/L Gibberellic acid (GA), pH 5.7. Germinated somatic embryos with fully developed cotyledons appeared in 4–6 weeks (Mathews et al., 1993; Ihemere, 2003; Msikita et al., 2006; Ihemere et al., 2008).

### Codon-optimization of *Chlamydomonas* *FEA1* for cassava

The codon-usage of the *Chlamydomonas* *FEA1* gene is extremely GC biased. Therefore, the *FEA1* gene was codon-optimized for expression in cassava. The Graphic Codon Usage Analyzer^1^ was used to optimize the codon-usage. Overlapping forward and reverse primers (Tables A1 and A2 in Appendix) for PCR re-assembly of gene fragments using 20–40-mer oligonucleotide primers were designed. One unit (U) of Platinum^®^ *Pfx* DNA polymerase (Invitrogen), plus 1× reaction buffer and 2.5 μM of overlapping primers were used in the PCR reaction. The DNA amplification was carried out for 55 cycles at 94°C for 5 min, 94°C for 30 s, 55°C for 30 s (annealing temperature), and 68°C for 40 s (extension temperature). A second PCR reaction was carried out using 2.5 μL from the first PCR reaction as template and 0.5 μM of the outer forward and reverse primers plus 1 U of Platinum^®^ *Pfx* DNA Polymerase (Invitrogen). DNA amplification was carried out for 30 PCR cycles at 94°C for 5 min, 94°C for 30 s, 55°C for 30 s (annealing temperature), 68°C for 1 min (extension temperature), and 68°C for 10 min (final extension temperature). The fidelity of the PCR product was confirmed by DNA sequencing analysis at The Ohio State University Plant Microbe Genomics Facility.

### Construction of Ti-plasmid binary vector

A modified pBI121 Ti-plasmid (Clontech) containing the patatin-*FEA1* gene insert (3DF* plasmid) was used for cassava transformation. The endogenous CaMV 35S promoter was substituted with the 1.0 kb potato patatin promoter to drive *FEA1* expression (AY485645) cloned into the *Hin*dIII and *Sma*I restriction sites (Rosahl et al., 1986). The *FEA1* gene was cloned downstream of the patatin promoter between the *Sma*I and *Sst*I restriction sites. The terminator for the *FEA1* gene is the *Agrobacterium* *nos* terminator (Bevan, 1984). The T-DNA also included the *npt*II gene conferring resistance to kanamycin and its analog paromomycin. The *npt*II gene was driven by *nos* promoter and had a 3′ *nos* terminator. This construct was given the name 3DF*. The 3DF* plasmid was transformed into *E. coli* and confirmed by PCR analysis using *FEA1* gene-specific primers. The forward primer was 5′-GCACAGTTAACC **CCCGGG**ATGTCTGTCGGATTTCTGGTCCTC-3′ and reverse primer 5′-CATGGA**GAGCTC**ACAGTATTACATTACAGCTCCTCTCCTCCA-3′ targeting the full-length *FEA1* gene. The codons in bold represent the restriction site for *Sma*I (forward primer) and *Sst*I (reverse primer). The concentration of templates was 100 ng and that of primers was 10 μM per 50 μL PCR reaction. The PCR conditions were as follows: 4 min at 94°C, 30 s at 94°C, 1 min 45 s at 58°C, and 1 min 40 s at 72°C, for 30 cycles. The product was again confirmed by DNA sequence analysis. 3DF* plasmid DNA (100 ng) was used to transform *Agrobacterium tumefaciens* strain LBA4404 from Invitrogen (Rockville, MD, USA). Colonies that were resistant to kanamycin and streptomycin were screened by PCR with *nptII* specific primers. The forward primer was 5′-CTTCGTGGCCGTGACCCGCGCGGC-3′ and the reverse primer was 5′-CCGAATTCATAGATGACCCGCGC-3′. DNA amplification was carried out for 30 PCR cycles at 94°C, 3 min; 30 s at 94°; 55°, 60 s (annealing temperature); 50 s at 72°C (extension temperature); and 72°C, 4 min. The concentration of templates was 100 ng and that of primers was 10 μM per 50 μL PCR reaction.

### Cassava transformation

Cassava transformation was carried out using young leaf lobes and cotyledons of germinated somatic embryos (Arias-Garzon, 1997). *Agrobacterium* suspension carrying the 3DF* plasmid was co-cultivated with cassava somatic embryo cotyledons on MS basal medium plus 100 μM acetosyringone for 2 days. The tissues were then transferred to cassava somatic embryogenesis induction media comprising MS salts containing 8 mg/L 2,4-D, 75 mg/L paromomycin, and 500 mg/L carbenicillin to eradicate *Agrobacterium* and to select for transformants. Antibiotic resistant somatic embryos were grown under a 12 h/day photoperiod at a light intensity of 50 μmol photons/m^2^/s grown at 28°C. Clumps of somatic embryos that formed after 4 weeks of culture were transferred to cassava germination medium containing 75 mg/L paromomycin and 500 mg/L carbenicillin for four more weeks. After germination, individual plantlets were transferred to cassava micro-propagation medium [MS salts plus 2% (w/v) sucrose, 0.04 mg/L benzylamino purine, 0.05 mg/L giberellic acid, 0.02 mg/L NAA, 1.0 mg/L thiamine-HCl, 100 mg/L myo-inositol, pH 5.7] without antibiotics for root induction. Wild-type cassava (TMS 60444) plants used for control experiments were regenerated from somatic embryos using the same protocol to regenerate transgenic plants but without antibiotic selection (Msikita et al., 2006; Ihemere et al., 2008).

### PCR and reverse transcriptase-PCR analysis

Genomic DNA was isolated from 100 mg leaf tissue from *in vitro* transgenic and wild-type cassava using a Qiagen Plant DNA Extraction Kit (Qiagen Inc., Valencia, CA, USA). The DNA was amplified by PCR using *FEA1*-specific forward primers *FEA1*F1 5′-GCACAGTTAACCCCCGGGATGTCTGTCGGATTT CTGGTCCTC-3′ and reverse primers *FEA1R1* 5′-CTAGCGGCGTCGTAGGCTTGGATGTTCAGGGTAGACAGTCTG-3′. The PCR conditions were as follows: 4 min at 94°C, 30 s at 94°C, 45 s at 58°C, and 30 s at 72°C for 30 cycles, then 72°C, 10 min. The PCR product (450 bp) was run on 0.8% (w/v) agarose gel. The concentration of templates was 100 ng and that of primers was 10 μM per 50 μL PCR reaction. Total RNA was extracted from *in vitro* root and leaf materials (100 mg) using a Qiagen Plant RNA Extraction Kit (Qiagen Inc., Valencia, CA, USA). The RNA was treated with 1.0 U DNase (Invitrogen, Carlsbad, CA, USA) for 15 min at room temperature to eliminate DNA contamination. The DNase was inactivated by treating with 25 mM EDTA followed by heat inactivation at 65°C. The first strand cDNA synthesis was carried out with 10 μg of total RNA using 1× reverse transcription buffer, 0.3 mM dNTP, 0.5 μg oligodT_(12–18)_ primers, and 200 U of SuperScript III reverse transcriptase (Invitrogen, Carlsbad, CA, USA). The mixture was incubated at 65°C for 5 min without reverse transcriptase followed by incubation at 50°C for 1 h with reverse transcriptase and 15 min of enzyme heat inactivation at 70°C. The cDNA was amplified by PCR using *FEA1*-specific forward primers *FEA1F1* 5′-GCACAGTTAACCCCC GGGATGTCT GTCGGATTTCTGGTCCTC-3′ and reverse primers *FEA1R1* 5′-CTAGCGGCGTCGTAGGCTTGGATGTTCAGGGTAGACAGTCTG-3′. The PCR conditions were as follows: 4 min at 94°C, 30 s at 94°C, 45 s at 58°C, and 30 s at 72°C for 30 cycles and 10 min at 72°C. The concentration of template was 100 ng and that of primers was 10 μM per 50 μL PCR reaction. The PCR product (450 bp) was run on a 0.8% (w/v) agarose gel. Control experiments included no cDNA product (negative control for genomic DNA contamination) and amplification of the *sbe1* gene (Forward primer 5′-GAACGATTGGCTAAGTTGGCC-3′ and reverse primers 5′-GAGGGCCATACAAAATCCCCT-3′. The PCR conditions for the *sbe1* gene were as follows: 5 min at 94°C, 30 s at 94°C, 45 s at 54°C, and 40 s at 72°C for 30 cycles and 10 min at 72°C. The *sbe1* RT-PCR product is a 650 bp fragment. The α-tubulin RT-PCR (control) was performed with the following primers: Forward primer 5′-TATATGGCCAAGTGCGATCCTCGACA-3′ and reverse primer 5′-TTACTCTTCATAATCCTTCTCAAGGG-3′ for leaf RNA (positive standard). The conditions for the PCR reactions were as follows: 5 min at 94°C, 30 s at 94°C, 45 s at 58°C, and 30 s at 72°C for 30 cycles and 72°C for 10 min. The concentration of templates was 100 ng and that of primers was 10 μM per 50 μL PCR reaction. The tubulin PCR reaction amplified a 400 bp product. The expression levels of all metal homeostasis genes were normalized to cassava α-tubulin expression levels in the corresponding tissues.

### Ferric chelate reductase assay

Stem cuttings from *in vitro* grown transgenic and wild-type cassava were grown in liquid MS media salts containing 27.8 mg/L FeSO_4_·7H_2_O supplemented with 20% (w/v) sucrose for 4 weeks. Afterward, the plants were transferred to liquid MS media containing 25 or 5 μM Fe (III)-EDTA considered iron-sufficient or iron-deficient media, respectively, for 2 days. The ferric chelate reductase activity of *in vitro* cassava roots was measured in 1.0 mL of assay media comprising 1× MS media, 5 mM MES (pH 5), 0.1 mM Fe (III)-EDTA, and 0.3 mM sodium bathophenanthrolinedisulfonic acid (BPDS). The roots were incubated in the assay media for 20 min in the dark, thereafter, the roots were removed from the assay media, blotted dry on Whatman filter paper, and weighed. The absorbance of the assay media was measured at 535 nm. The rate of ferric reduction was calculated as μmol Fe (II)-BPDS per gram fresh weight per minute (Robinson et al., 1997). The molar extinction coefficient for Fe (II)-BDPS at 535 nm was 221,401/M/cm (Bruggemann et al., 1993).

### ICP-MS analysis of metal content

Three replicates of 1-month-old *in vitro* cassava plantlets with well-established roots were transferred to the greenhouse for the production of tuberous roots used for elemental ICP-MS measurements of iron. The plantlets (triplicates) were initially maintained in a growth chamber at 28°C at a light intensity of 50 μmol photons/m^2^/s for a week prior to transfer to soil in the greenhouse. Plantlets were then transferred to 11″ × 12″ plastic pots filled with Scott’s MetroMix soil (1.2% iron; The Scott’s Company, Marysville, OH, USA) after 30 days growth in the greenhouse. Tuberous roots, leaves, and stems were harvested from 6-, 9-, and 12-month-old plants for elemental ICP-MS analysis at the Ohio State University OARDC STAR Lab. After harvest the leaves, stems, and tuberous roots were separated, weighed, and washed for 5 min in prewashed glass beakers in the following solution: 400 mL de-ionized water, 400 mL 1.0 M MgSO_4_, with 5.0 mM Na dithionite (Na hydrosulfite), followed by washing with 400 mL de-ionized water. The plant tissues were then placed in paper bags for drying in an incubator at 80°C. The samples were weighed every 2 days until a constant dry weight was obtained. Each treatment had a minimum of three replicates. The *in vitro* fibrous and greenhouse-grown tuberous roots were prepared for ICP-MS analysis by removing all agar and loose dirt and washing the roots well in running water. The roots were then washed separately with 200 mL de-ionized water in prewashed glass beakers for 5 min. This was followed by a wash with 200 mL of 1.0 M MgSO_4_, with 5.0 mM Na dithionite (Na hydrosulfite) for 5 min. The samples were then rinsed with 200 mL de-ionized water for 5 min. The peels of tuberous roots were removed with a plastic knife. Separate plastic knifes were used to cut cortex pieces, approximately 1 g each. The samples were dried in Flexi-Dry freeze dryer (SP Industries, Warminster, PA 18974, USA) until a constant weight was attained. Tuberous roots, leaves, and stems were harvested from 6-, 9-, and 12-month-old plants for elemental ICP-MS analysis at the Ohio State University OARDC STAR Lab.

### Cassava database screening and mining

Domain search for iron metabolism genes was executed on the website of ESTIMA database at UIUC.^2^ Key words such as “iron transporters,” “NRAMP,” “YSL,” “Ferritin,” “ZIP/IRT,” “FRO” were used as queries. Two types of EST libraries were queried including, CV01: normalized library from cassava control; constructed from five samples: mature leaf and petiole, young leaf and apical meristem, root, tuber and tuber peel, young leaf and apical meristem midnight, and CV02: normalized library from cassava under water stress; constructed from four samples: young leaf and apical meristem, mature leaf and petiole, root, tuber and tuber peel. Sequences from the ESTs were used as queries to search the annotation database of cassava genome^3^ and 11 genes were identified and considered for further analysis.

### Phylogenetic analysis and sequence alignment

All sequences used in this study were aligned using ClustalW (Chenna et al., 2003) 2.0 with default settings. The aligned sequences were shaded using GeneDoc 2.6.02 (Nicholas and Nicholas, 1997) and copied into an RTF file for further annotation. Sequences were compared according to conserved amino acid numbers. The phylogenetic tree was displayed and annotated using MEGA software version 4 (Tamura et al., 2007). Bootstrap analysis was performed using 1,000 replicates. The unrooted phylogenetic tree was drawn based on the protein sequences with neighbor-joining method (Saitou and Nei, 1987).

### RNA extraction and reverse transcription

Total RNA from roots, stem, and leaves of wild type and two *FEA1* transgenics 3DF*6 and 3DF*9 (6–8 weeks *in vitro* old plants) was extracted using the RNA-easy kit from Qiagen Inc. (Valencia, CA, USA) according to the manufacturer’s instructions. To remove contaminating genomic DNA, RNAs were treated with the DNAase I (Promega, Madison, WI, USA) according to the manufacturer’s instructions. The concentrations of RNAs were assessed using a Nanodrop-2000C (Thermo-scientific, Wilmington, DE, USA) according to the manufacturer’s instructions. The structural integrity of the RNAs was checked with non-denaturing agarose gel and ethidium bromide staining. DNase-treated RNA samples (0.5 μg) were reverse transcribed with an anchored oligo (dT) primer and 200 U superscript II reverse transcriptase (Invitrogen, Carlsbad, CA, USA) in a volume of 20 μL according to the manufacturer’s instructions.

### Semi-quantitative PCR

PCR reactions were carried out with gene-specific primers (Table 1). Additional reaction components were: 10 mM polymerase buffer, 1 mM dNTPs, 0.1 U *Taq polymerase*, and 10 μM specific primers. The conditions for the PCR were as follows: 5 min at 94°C, 30 s at 94°C, 30 s at 55°C, and 1 min at 72°C for 35 cycles and 72°C for 10 min. The concentration of templates was 100 ng per 50 μL PCR reaction. Amplified products were visualized on a 1% TAE agarose gel containing ethidium bromide. Bands were photographed using the Quantity One 4.5.1 Chemidoc EQ™ Software System (Bio-Rad, CA, USA).

**Table 1:** **Forward and reverse primers for semi-quantitative PCR amplification**.

Gene	Forward primer (5′–3′)	Reverse primer (5′–3′)
*MeFRO2*	GGCTGAGAAGGTTCCAAATAATG	TTTATAATTTTGACTAATAAATATAAGCAGGC
*MeIRT1*	GTGAAAATATGGTGCTTGATTTTG	TACATAGATGAATACTCCACATGCTAG
*MeIRT2*	GGCTATTGGGATAGTTATAGATGCA	AAACTGCATTTCATTTGGCTATTT
*MeIRT6*	AGGAATCTCTTTAGGTGCTTCTCA	ACAAAGGAGGGTTGGTCCTAA
*MeNRAMP2*	TCAATGGTTACCTATTGGTCGAC	TCCTACTGACACTTACTAACATAAGCTACA
*MeNRAMP3*	CTTTATGGGCTGGGGTTATTATC	AAGAAGAGGAATAAGTGCAAAAGG
*MeYSL1*	CAATAAGAAATGGTCAAGATTCATTC	ATTGAAAATACTTATCTAGAGGGATCTACA
*MeYSL2*	AACCTGGGATTGGTTGGATG	GAAATACAGTATCTTGAGAAAATTGTAGAG
*MeFER1*	GCTTTATATGCTATGGAGTTAGCATTG	CAAAACAGCTGCCTGCTTT
*MeFER3*	TACCCATTGCTTCCATTGC	TTGGCAAGACCCTTGAGAG
*MeFER8*	TTCTTCTTCTTCTCTCTCAGCTCTC	TGCAGAAGCATCTGATCAAAGT
*MeTubulin*	GATCCTACTGGGAAGTACATTGG	CTGCATTCTCCACCAACTGA

### Real-time PCR analysis

Total RNA from roots, stems, and leaves of wild-type and *FEA1* transgenic cassava (6–8 weeks *in vitro* old plants) was isolated using the RNA-easy kit from Qiagen Inc. (Valencia, CA, USA) according to the manufacturer’s instructions. Real-time quantitative RT-PCR was carried out using an ABI – Step One Plus (Applied Biosystems, Foster City, CA, USA) using PerfeCTa™ SYBR^®^ Green FastMix™ (ROX dye; Quanta Biosciences, Gaithersburg, MD, USA) according to manufacturer’s instructions. Reactions were carried out with RNA of 50–100 ng/μL in a final volume of 20 μL. DNase-treated RNA samples were reverse transcribed with an anchored oligo (dT) primer and 200 U superscript II reverse transcriptase (Invitrogen, Carlsbad, CA, USA) in a volume of 20 μL according to the manufacturer’s instructions. All the primers were designed using the Primer Express software following the manufacturer’s guidelines.^4^ Primers are listed in Table 2. Alpha tubulin expression was used as a constitutive control to normalize iron homeostasis gene expression. PCR cycling conditions comprised an initial denaturation holding stage at 95°C for 10 min, followed by 40 cycles of cycling stage at 95°C for 15 s, 55°C for 15 s, and 72°C for 30 s and final melt curve stage 95°C for 15 s, 60°C for 1 min, and 95°C for 15 s. For each sample, reactions were set up in quadruplicates and two biological experiments were done to ensure the reproducibility of the results. The quantification of the relative transcript levels was performed using the comparative *C*_T_ (threshold cycle) method (Livak and Schmittgen, 2001).

**Table 2:** **Forward and reverse primers for quantitative PCR (real-time) amplification**.

Gene	Forward primer (5′–3′)	Reverse primer (5′–3′)
*MeFRO2*	GTTGGATTGTGGCATCTGTG	ATTATCACCCCCACATCGAC
*MeIRT6*	GGTATGGGACTTGGTGGTTG	AAAATTCCAGCTGAGGCTGA
*MeYSL1*	CCCAATGGCAATGGCTATAC	TATTCCATCGCCACAGATCA
*MeFER1*	AACTGGAACTGGGTTTGTGG	CATGGCATGGTACACGTAGG
*MeFER3*	TGATGTGCAGTTGGGTGATT	GCAACAACTGCCTCTTCCTC
*MeTubulin*	GTGGAGGAACTGGTTCTGGA	TGCACTCATCTGCATTCTCC

## Results

### Generation and molecular analysis of transgenic cassava plants expressing the *FEA1* gene

**Figure 1:**
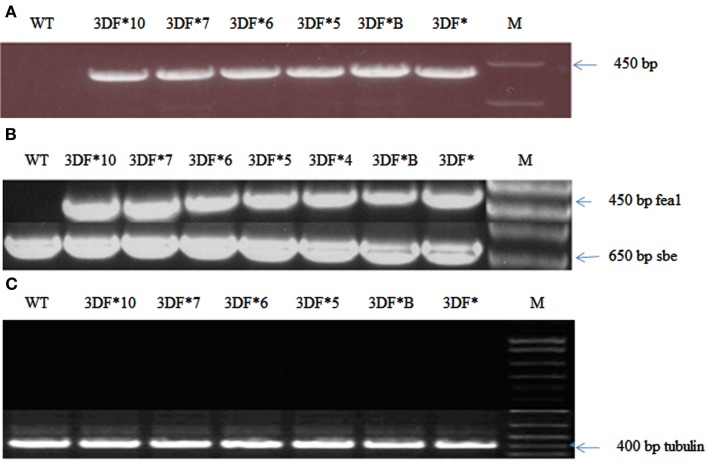
**Confirmation of cassava transformants with modified *FEA1* gene**. **(A)** PCR amplification of the *FEA1* gene in transgenic cassava. WT, wild-type cassava; 3DF*, 3DF*B, 3DF*5, 3DF*6, 3DF*7, and 3DF*10 are transgenic cassava. **(B)** Analysis of *FEA1* gene expression: **(A)** RT-PCR analysis of *FEA1* gene expression in roots of transgenic and wild-type cassava plants. The cassava *sbe1* gene was used as loading control. **(C)** RT-PCR analysis of *FEA1* gene expression in leaves of transgenic and wild-type cassava plants. Loading control for **(B)** is cassava α-tubulin gene.

The cassava cultivar TMS 60444 was transformed with a codon-optimized version of the iron assimilatory gene *FEA1* from *C. reinhardtii* whose expression was driven by the patatin promoter. We have previously shown that the patatin promoter drives high levels of gene expression in cassava roots (Siritunga and Sayre, 2003; Ihemere et al., 2006). A total of 50 independent paromomycin-resistant cassava lines were generated using *Agrobacterium* Ti-plasmid mediated transformation. PCR screening for the presence of the *FEA1* gene indicated that 25 plants or 50% of the putative transgenics were PCR positive. We focused our analysis on six independent cassava lines which were representative of the transgenic population based on PCR and RT-PCR confirmation of *FEA1* gene expression (discussed below). PCR and RT-PCR analyses to confirm the integration and expression of the *FEA1* gene are shown in Figure 1A. To determine the tissue-specific expression of the *FEA1* gene, we performed semi-quantitative RT-PCR analysis of 6-week-old *in vitro* leaves and roots of transgenic and wild-type cassava. There was detectable expression of the *FEA1* gene in the roots of all seven independent transgenic lines studied (Figure 1B). As shown in Figure 1C, there was no detectable expression of the *FEA1* gene in leaves of transgenic plants consistent with previous observations using patatin-driven gene constructs (Siritunga and Sayre, 2004; Ihemere et al., 2006).

### *FEA1* expression in cassava enhances iron accumulation in transgenic roots

**Figure 2:**
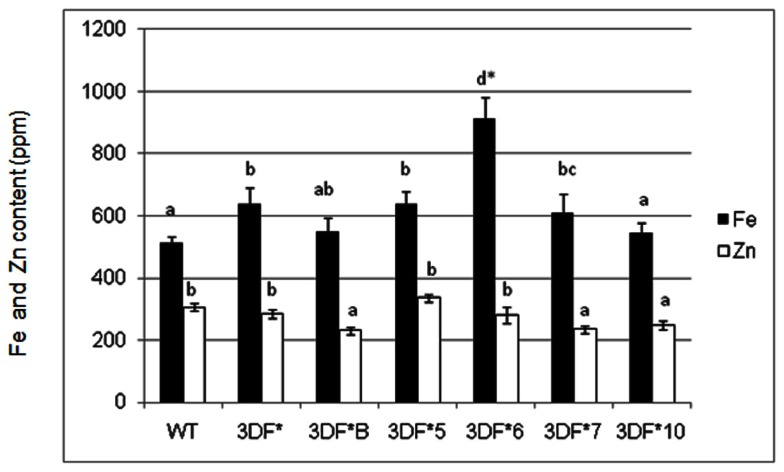
**Iron and zinc content of 6-week-old *in vitro* fibrous roots of *FEA1* transgenic and wild-type cassava plants**. The results represent the mean of three replicates (±standard deviation). White bars represent zinc and black bars represent iron. WT, wild type; 3DF*, 3DF*B, 3DF*4, 3DF*5, 3DF*6, 3DF*7, and 3DF*10 are *FEA1* transgenic cassava plants. *Numbers with same letters show no statistical difference, different letters mean statistical difference at 95% confidence level.

Iron and zinc content in transgenic and wild-type cassava plants of various ages was measured by ICP-MS analysis of freeze-dried or oven-dried plant material. Iron uptake takes place predominantly via iron transporters located in the root hair of fibrous roots and presumably is absent in mature storage roots lacking roots hairs (Frossard et al., 2000). To determine the relative extent of iron assimilation in transgenic and wild-type cassava roots, we measured the iron content of 6-week-old (*in vitro*) fibrous roots. Iron levels of 6-week-old (*in vitro*) fibrous roots of *FEA1* transgenic cassava plants ranged from 544 to 914 ppm while the wild-type cassava roots had an iron content of 514 ppm, or 80% less than the highest iron accumulating transgenic lines (Figure 2). Importantly, the Fe content of the highest iron accumulating transgenic lines was significantly higher than the wild-type roots (Figure 2). In contrast, there were no significant differences in the zinc levels for either roots and leaves between the transgenic and wild-type plants. Zinc levels of 6-week-old (*in vitro*) fibrous roots of *FEA1* transgenic cassava plants ranged from 231 to 284 ppm while the wild-type cassava roots had an iron content of 307 ppm (Figure 2). These results are consistent with the *C. reinhardtii* *FEA1* gene being specific for Fe transport (Rubinelli et al., 2002; Narayanan et al., 2011; Leyva-Guerrero et al., 2012).

**Figure 3:**
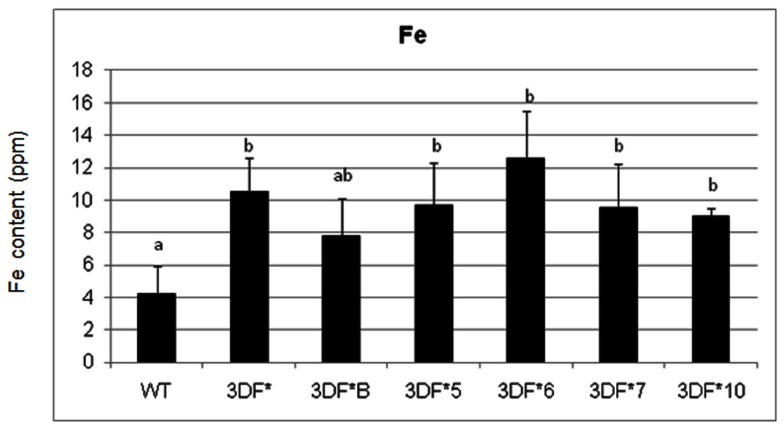
**Iron content of greenhouse-grown 6-month-old roots of *FEA1* transgenic and wild-type cassava plants**. The results represent the mean of three replicates (±standard deviation). WT, wild type TMS 60444 cassava; 3DF*, 3DF*B, 3DF*4, 3DF*5, 3DF*6, 3DF*7, and 3DF*10 are *FEA1* transgenic cassava plants. Iron content is expressed as ppm = mg Fe/kg dry weight. *Numbers with same letters show no statistical difference, different letters indicates statistically significant difference at 95% confidence level.

In contrast to fibrous roots, 6-month-old tuberous roots are rapidly filling with starch, lack root hairs and presumably are not actively taking up iron directly from the soil. Iron concentrations in 6-month-old wild-type tuberous roots decreased over 100-fold relative to fibrous roots (Figure 3). In transgenic plants the root iron levels decreased 70-fold but decreased to a lesser extent than in wild-type roots over the same time period (6 weeks to 6 months). The highest iron accumulating transgenic line (3DF*6) showed threefold increased iron levels (12.6 ppm) when compared with the wild-type roots (4.2 ppm; Figure 3). The other transgenic lines studied had iron contents of approximately 10 ppm or 2.5 times more iron than wild-type roots (Figure 3).

**Figure 4:**
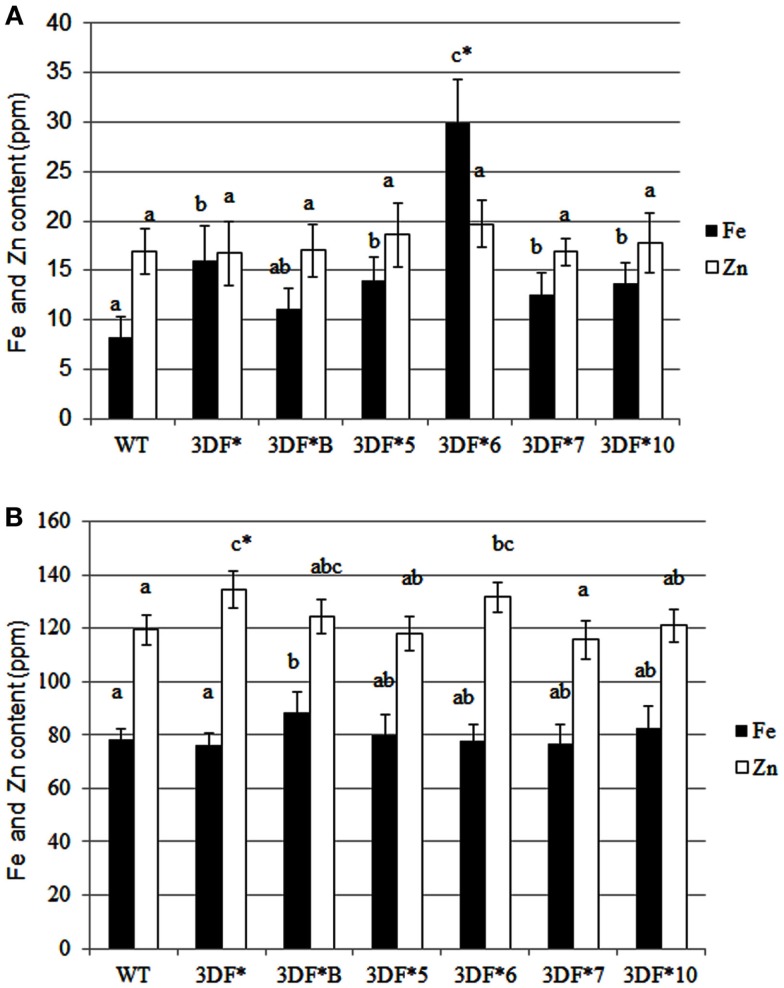
**Iron and zinc content of 9-month-old greenhouse-grown storage roots and leaves of *FEA1* transgenic and wild-type plants**. White bars represent zinc and black bars represent iron. **(A)** Iron and zinc content of cassava storage roots. WT, wild type; 3DF*, 3DF*B, 3DF*5, 3DF*6, 3DF*7, and 3DF*10 are *FEA1* transgenic cassava plants. **(B)** Iron and zinc content of leaves from 9-month-old greenhouse-grown plants of *FEA1* transgenic and wild-type cassava plants. The results represent the mean of three replicates (±standard deviation). *Numbers with same letters show no statistical difference, different letters mean statistical difference at 95% confidence level.

Following 9 months growth in the greenhouse, the highest Fe-accumulating transgenic line, 3DF*6, had Fe levels of 30 ppm, or a 2.4-fold increase relative to 6-month-old roots, while wild-type cassava roots had iron levels of 8.25 ppm, about a twofold increase relative to 6-month-old roots (Figure 4A). At 9 months, the Fe concentration in the highest iron accumulating transgenic line (3DF*6) was threefold greater than wild-type cassava roots (Figure 4A). In contrast, the iron levels in leaf tissues of 9-month-old transgenic plants were not significantly different from those of wild-type leaves. Transgenic plants had leaf iron levels ranging between 70 and 88 ppm Fe while wild-type leaves had iron levels of 60 ppm. In wild-type plants, leaf iron concentrations were sixfold greater than the iron levels of roots suggesting that leaves act as a primary sink for iron consistent with the need for iron for proteins involved in photosynthetic electron transfer (Figure 4B).

**Figure 5:**
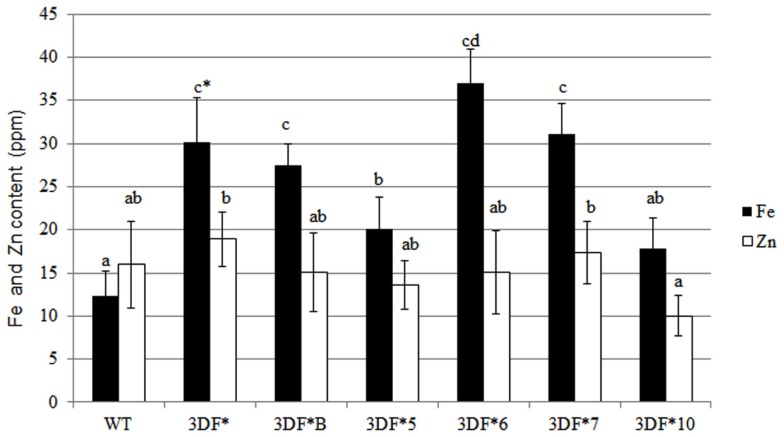
**Iron and zinc content of 12-month-old storage roots of greenhouse-grown *FEA1* transgenic and wild-type cassava plants**. White bars represent zinc and black bars represent iron. WT, wild type; 3DF*, 3DF*B, 3DF*5, 3DF*6, 3DF*7, and 3DF*10 are *FEA1* transgenic cassava plants. The results represent the mean of three replicates (±standard deviation). *Numbers with same letters show no statistical difference, different letters mean statistical difference at 95% confidence level.

We also measured root iron levels at the time (12 months after planting) roots are typically harvested in the field. At 12 months of age, root iron concentrations increased again relative to 9-month-old plants. The highest accumulator, 3DF*6, had root iron levels of 36 ppm while wild-type roots had threefold lower iron levels (12 ppm; Figure 5). Storage root iron levels in the other transgenic cassava lines ranged between 17 and 30 ppm (Figure 5). The increase in iron concentration in older roots may reflect differential rates of biomass and iron accumulation in different aged roots. Importantly, there was no significant difference in Zn levels between wild-type or transgenic roots or leaves at any stage in the growth of the plants that we assessed (Figures 2–5). These results re-confirm earlier observations that the FEA1 protein does not facilitate uptake of zinc in contrast to other iron transporters present in plants (Henriques et al., 2002; Vert et al., 2002; Palmer and Guerinot, 2009).

**Figure A1:**
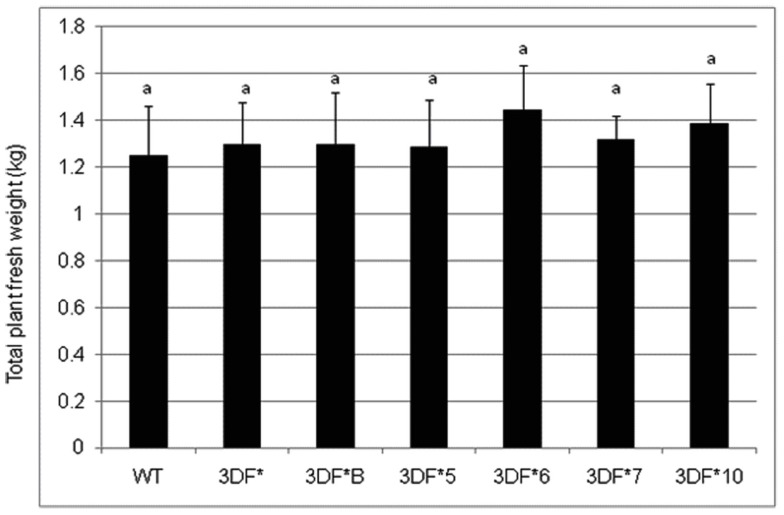
**The total fresh weight of *fea1*-transformed cassava and wild-type (WT) cassava plants**. The plants were grown in the greenhouse for 6 months. Three independent replicates of wild-type and transformed plants were used in the analysis. *Numbers with same letters show no statistical difference, different letters mean statistical difference at 95% confidence level.

**Figure A2:**
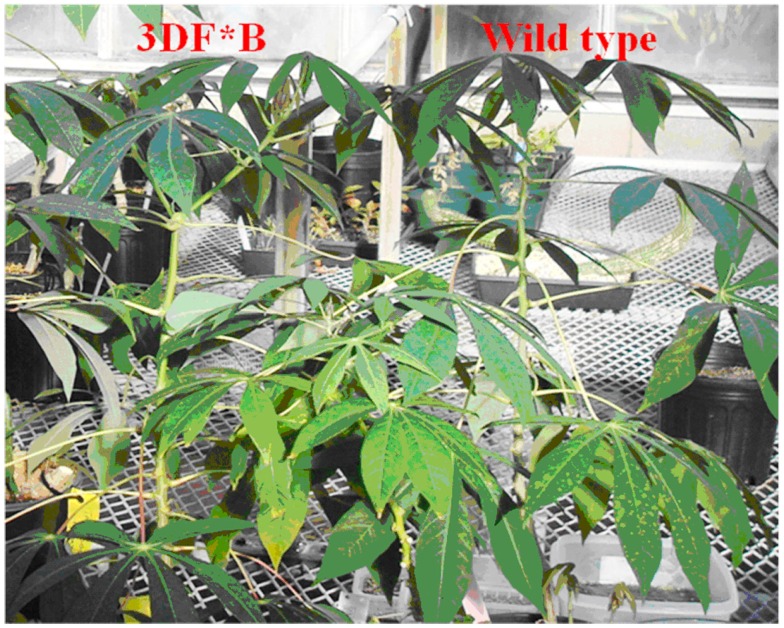
Phenotypic comparison of 4-month-old transgenic FEA1 cassava (3DF*B) and wild-type cassava. The plantlets were initially maintained in a greenhouse growth chamber at 28°C at a light intensity of 50 μmol photons/m^2^/s. Plantlets were transferred to 11″ × 12″ plastic pots filled with Scott’s MetroMix soil (The Scott’s Company, Marysville, OH, USA) after 30 days growth in the greenhouse growth chamber.

The effect of *FEA1* gene on the overall growth of cassava plants was also studied after 6 months growth in the greenhouse. We observed no significant difference in the total top and bottom fresh weight between the wild-type and the FEA1 transgenic cassava plants. Whole plant fresh weight ranged from 1.2 to 1.4 kg (Figure A1 in Appendix). We also observed no morphological differences between wild-type and transgenic plants throughout the study (Figure A2 in Appendix). These results demonstrate that expression of the FEA1 protein in cassava did not have any detrimental effect on yield or growth of the plant.

### *FEA1* transgenic cassava plants have reduced ferric chelate reductase activity

**Figure 6:**
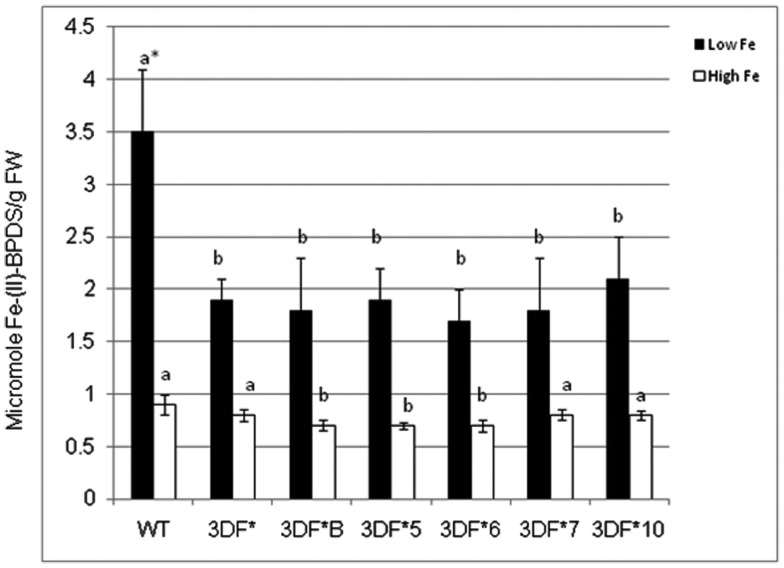
**Ferric chelate reductase activity of *FEA1* transgenic and wild-type cassava plants**. Stem cuttings from *in vitro* grown transgenic (3DF*, 3DF*B, 3DF*5, 3DF*6, 3DF*7, and 3DF*10) cassava and wild-type cassava were grown in MS media salts supplemented with 20% (w/v) sucrose for 4 weeks. Afterward, the plants were transferred to liquid MS media containing either 25 or 5 μM Fe(III)-EDTA for 2 days. White bars represent high Fe and black bars represent low Fe treatment. *Numbers with same letters show no statistical difference, different letters mean statistical difference at 95% confidence level.

To determine if expression of the *FEA1* gene in fibrous roots actively transporting and storing iron reduced the demands for iron uptake by endogenous iron transport systems, we compared ferric chelate reductase activity under iron deficient and sufficient growth conditions. Ferric chelate reductase activity is typically elevated in Strategy I plants grown under iron-deficient conditions and is an indirect indicator of the iron status of the plant (Robinson et al., 1999; Connolly et al., 2003; Palmer and Guerinot, 2009). Plants deficient in iron have higher ferric chelate reductase activity (Robinson et al., 1997; Vasconcelos et al., 2006; Palmer and Guerinot, 2009; Li et al., 2011). It was our hypothesis that transgenic plants expressing the *FEA1* gene would have reduced iron demands due to facilitated uptake of iron. To test this hypothesis, we measured ferric chelate reductase activity in wild-type and transgenic plants grown under iron-deficient growth conditions. Plants were initially grown (*in vitro*) in iron-sufficient [25 μM (FeIII) EDTA] medium for 4 weeks and then transferred to iron-deficient [5 μM (FeIII) EDTA] medium for 2 days prior to measuring ferric chelate reductase activity. We observed that the ferric chelate reductase activity of fibrous roots was significantly lower in transgenic cassava expressing the *FEA1* gene than in wild-type cassava roots grown under iron-deficient conditions. The ferric chelate reductase activity of wild-type cassava roots was 3.5 μmol Fe (II)-BPDS/gfw/min. while the ferric chelate reductase activity of *FEA1* transgenic cassava plants was 50–60% lower (Figure 6). In contrast, the ferric reductase activity of wild-type and transgenic *FEA1* cassava plants grown under iron-sufficient conditions was essentially identical [0.7–0.9 μmol Fe(II)-BPDS/gfw/min] and substantially lower than that observed in plants grown under iron-deficient conditions (Figure 6).

### Altered expression of genes involved in iron homeostasis in cassava

**Figure 7:**
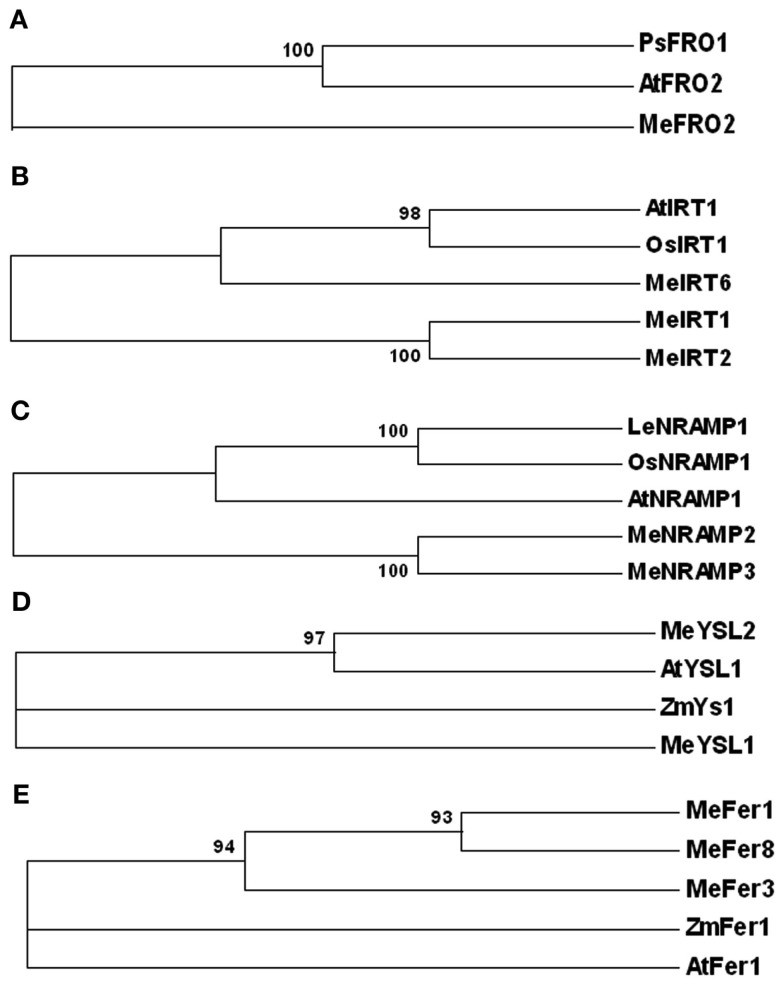
**Phylogenetic relationships of *Manihot esculenta* metal transporters and storage proteins. Bootstrap values from 1,000 replicates are indicated at each node**. **(A)** FRO family; Species designations are as follows: At, *A. thaliana* (AtFRO2); Ps, *P. sativum* (PsFRO1); Me, *M. esculenta* (MeFRO2). **(B)** IRT family; Species designations are as follows: At, *A. thaliana* (AtIRT1); Os, *O. sativa* (OsIRT1); Me, *M. esculenta* (MeIRT1, MeIRT2, and MeIRT6). **(C)** NRAMP family; Species designations are as follows: At, *A. thaliana* (AtNRAMP1); Os, *O. sativa* (OsNRAMP1); Le, *Lycopersicum esculentum* (LeNRAMP1); Me, *M. esculenta* (MeNRAMP2, MeNRAMP3). **(D)** YSL family; Species designations are as follows: At, *A. thaliana* (AtYSL1); Zm, *Zea Mays* (ZmYS1); Me, *M. esculenta* (MeYS1, MeYSL2). **(E)** Ferritin family. Species designations are as follows: At, *A. thaliana* (AtFER1); Zm, *Zea Mays* (ZmFER1); Me, *M. esculenta* (MeFER1, MeFER3, and MeFER8).

**Figure A3:**
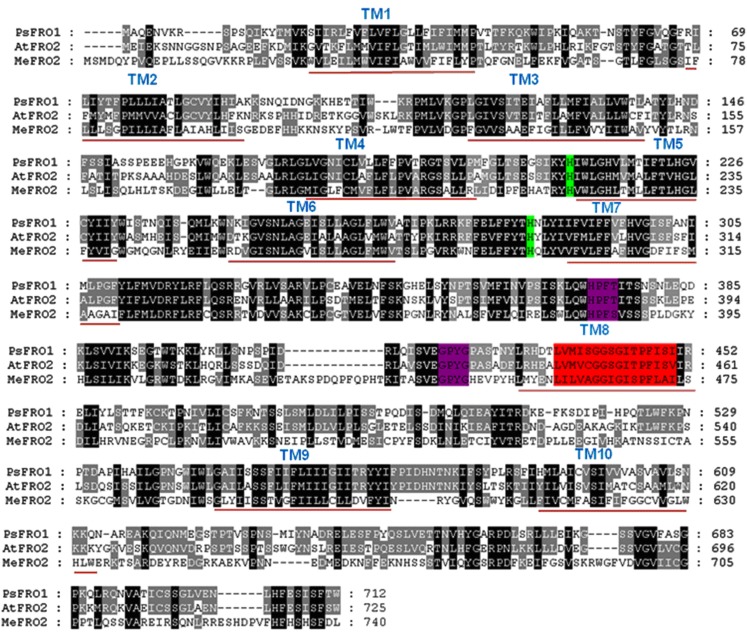
**Comparison of the putative amino acid sequences of MeFRO and other plant FRO proteins**. Identical and conservative amino acids are shaded in black and gray, respectively. Dashes indicate gaps. Ten transmembrane domains (TM) were identified using TMHMM and are shown as red lines below the sequences. Conserved histidines involved in heme binding are highlighted green, the conserved FAD-binding motif (HPFT) and the NAD-binding motif (GPyG) are highlighted in purple. The oxidoreductase signature sequence are highlighted in red. Species designations are as follows: At, *A. thaliana* (AtFRO2); Ps, *P. sativum* (PsFRO1); Me, *Mannihot esculenta* (MeFRO2).

**Figure A4:**
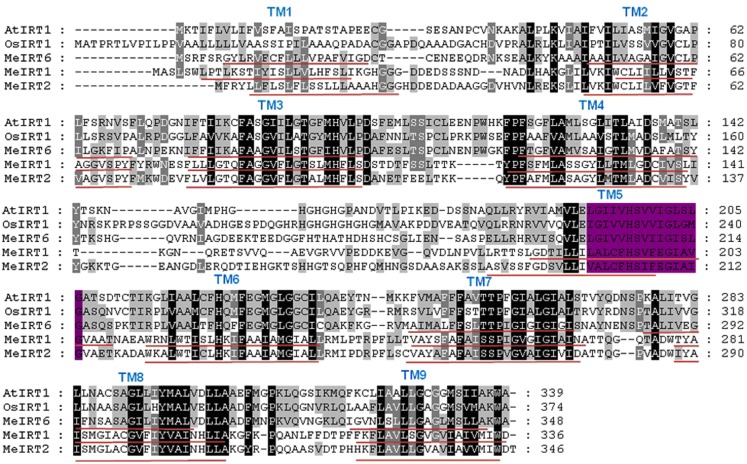
**Comparison of the putative amino acid sequences of MeIRT1 and other plant IRT1 proteins**. Identical and conservative amino acids are shaded in black and gray, respectively. Dashes indicate gaps. Nine transmembrane domains (TM) were identified using TMHMM and are shown as red lines below the sequences. The highly conserved motif for ZIP proteins in the fifth TM is highlighted in purple. Species designations are as follows: At, *A. thaliana* (AtIRT1); Os, *O. sativa* (OsIRT1); Me, *Mannihot esculenta* (MeIRT1, MeIRT2, and MeIRT6).

**Figure A5:**
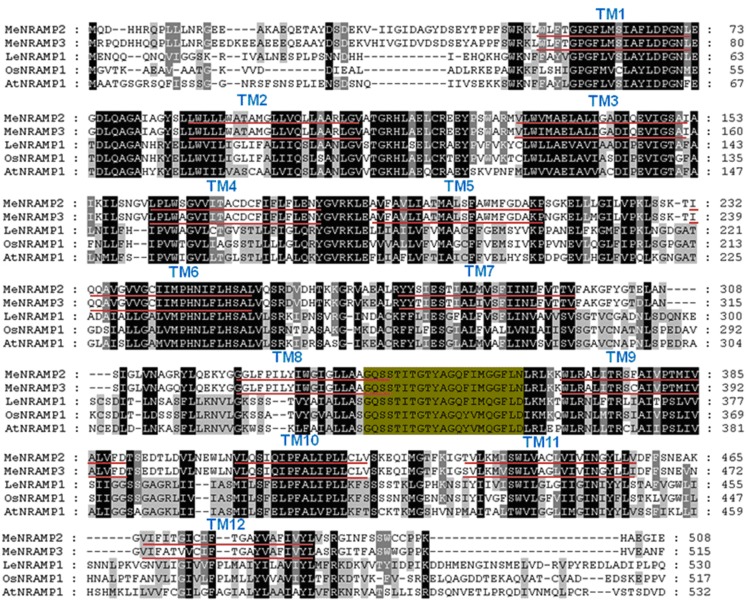
**Comparison of the putative amino acid sequences of MeNRAMP and other plant NRAMP proteins**. Identical and conservative amino acids are shaded in black and gray, respectively. Dashes indicate gaps. Twelve transmembrane domains (TM) were identified using TMHMM and are shown as red lines below the sequences. The consensus transport motif (CTM) is highlighted in green. Species designations are as follows: At, *A. thaliana* (AtNRAMP1); Os, *O. sativa* (OsNRAMP1); Le, *Lycopersicum esculentum* (LeNRAMP1); Me, *Mannihot esculenta* (MeNRAMP2, MeNRAMP3).

**Figure A6:**
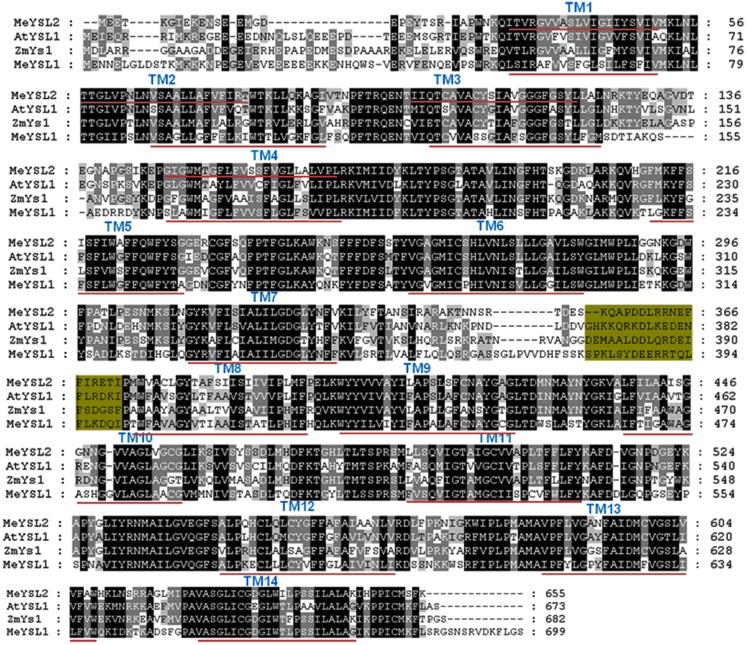
**Comparison of the putative amino acid sequences of MeYSL and other plant YSL proteins**. Identical and conservative amino acids are shaded in black and gray, respectively. Dashes indicate gaps. Fourteen transmembrane domains (TM) were identified using TMHMM and are shown as red lines below the sequences. Green shaded sequences represent the sequence determining structural property and substrate specificity of YS1/YSL transporters. Species designations are as follows: At, *A. thaliana* (AtYSL1); Zm, *Zea Mays* (ZmYS1); Me, *Mannihot esculenta* (MeYS1, MeYSL2).

**Figure A7:**
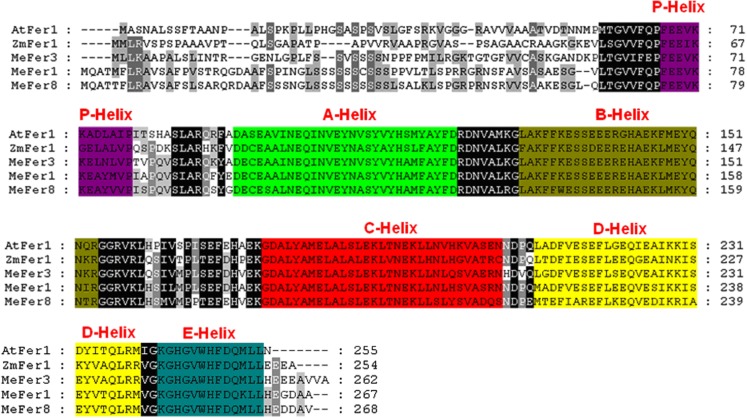
**Comparison of the putative amino acid sequences of cassava ferritin and other plant ferritin proteins**. Identical and conservative amino acids are shaded in black and gray, respectively. Dashes indicate gaps. Different helixes of ferritin protein are shaded as indicated. Species designations are as follows: At, *A. thaliana* (AtFER1); Zm, *Zea Mays* (ZmFER1); Me, *Mannihot esculenta* (MeFER1, MeFER3, and MeFER8).

To gain further insight into the control of iron assimilation and homeostasis in transgenic and wild-type cassava, we characterized the expression of variety of genes involved in metal uptake and homeostasis. Preliminary analysis of cassava library identified 11 genes potentially involved in maintaining iron homeostasis. To examine in detail the phylogenetic relationship of these members, the aligned protein sequences were used to construct the joint unrooted phylogenetic tree (Figure 7, Figures A3–A7 in Appendix). For each protein, the number of amino acids, conserved features, and homology with other known proteins are outlined in Table 3. Ferric reductase oxidase (FRO2) which encodes root ferric chelate reductase, and IRT1, which encodes the ferrous iron transporter, play a major role in primary acquisition of iron from soil to root (Guerinot, 2000; Maser et al., 2001). One member of FRO2 (MeFRO2) and three members of the IRT1 gene family were identified in cassava (MeIRT1, MeIRT2, and MeIRT6). Members of the Natural Resistance Associated Macrophage Protein (NRAMP) family of transporters also have key roles in mobilizing vacuolar stores of Fe during germination (Bereczky et al., 2003). In cassava, we identified two members of NRAMP family (MeNRAMP2 and MeNRAMP3). Members of the Yellow Stripe-Like (YSL) family of Fe (II)–nicotianamine transporters have been proposed to function as key mediators for long-distance Fe circulation/distribution within the plant (Curie et al., 2001; Connolly and Guerinot, 2002). Cassava, also had two members of YSL family (MeYSL1 and MeYSL2). Finally, the plastidic protein ferritin functions both in iron storage and iron detoxification in most organisms. Three ferritin gene family members were identified in cassava (MeFER1, MeFER2, and MeFER6).

**Table 3:** **Representative metal homeostasis genes in cassava**.

Protein	No. of amino acids	Conserved features	Homology with other known proteins	
			Similarity (%)	Identity (%)
MeFRO2	740	FAD-binding motif (HPFT); NADPH binding motif (GPYG); NADPH oxido reductases (MISGGSGITPFISI)	AtFRO2 (53)	AtFRO2 (32)
MeIRT1	336	Conserved motif (LGIIVHSVVIGLSL)	AtIRT1 (40)	AtIRT1 (38)
			OsIRT1 (40)	OsIRT1 (22)
MeIRT2	346	Conserved motif (LGIIVHSVVIGLSL)	AtIRT1 (44)	AtIRT1 (23)
			OsIRT1 (40)	OsIRT1 (26)
MeIRT6	348	Conserved motif (LGIIVHSVVIGLSL)	AtIRT1 (62)	AtIRT1 (47)
			OsIRT1 (62)	OsIRT1 (46)
MeNRAMP2	508	Consensus Transport motif (GQSSTITGTYAGQYVMQGFLD)	LeNRAMP1 (54)	LeNRAMP1 (36)
			OsNRAMP1 (53)	OsNRAMP1 (35)
			AtNRAMP1 (54)	AtNRAMP1 (54)
MeNRAMP3	515	Consensus Transport motif (GQSSTITGTYAGQYVMQGFLD)	LeNRAMP1 (63)	LeNRAMP1 (43)
			OsNRAMP1 (62)	OsNRAMP1 (42)
			AtNRAMP1 (62)	AtNRAMP1 (42)
MeYSL1	699	Substrate specificity (DEMAALDDLQRDEIFSDGSF)	AtYSL (68)	AtYSL (50)
			ZmYS1 (66)	ZmYS1 (50)
MeYSL2	655	Substrate specificity (DEMAALDDLQRDEIFSDGSF)	AtYSL (80)	AtYSL (66)
			ZmYS1 (75)	ZmYS1 (61)
MeFER1	267	Helixes (P, A, B, C, D, E)	AtFER1 (74)	AtFER1 (64)
			ZmFER1 (72)	ZmFER1 (62)
MeFER3	262	Helixes (P, A, B, C, D, E)	AtFER1 (72)	AtFER1 (62)
			ZmFER1 (74)	ZmFER1 (62)
MeFER8	268	Helixes (P, A, B, C, D, E)	AtFER1 (68)	AtFER1 (55)
			ZmFER1 (65)	ZmFER1 (54)

### Differential expression of metal-regulated genes in *FEA1* transgenic plants

**Figure 8:**
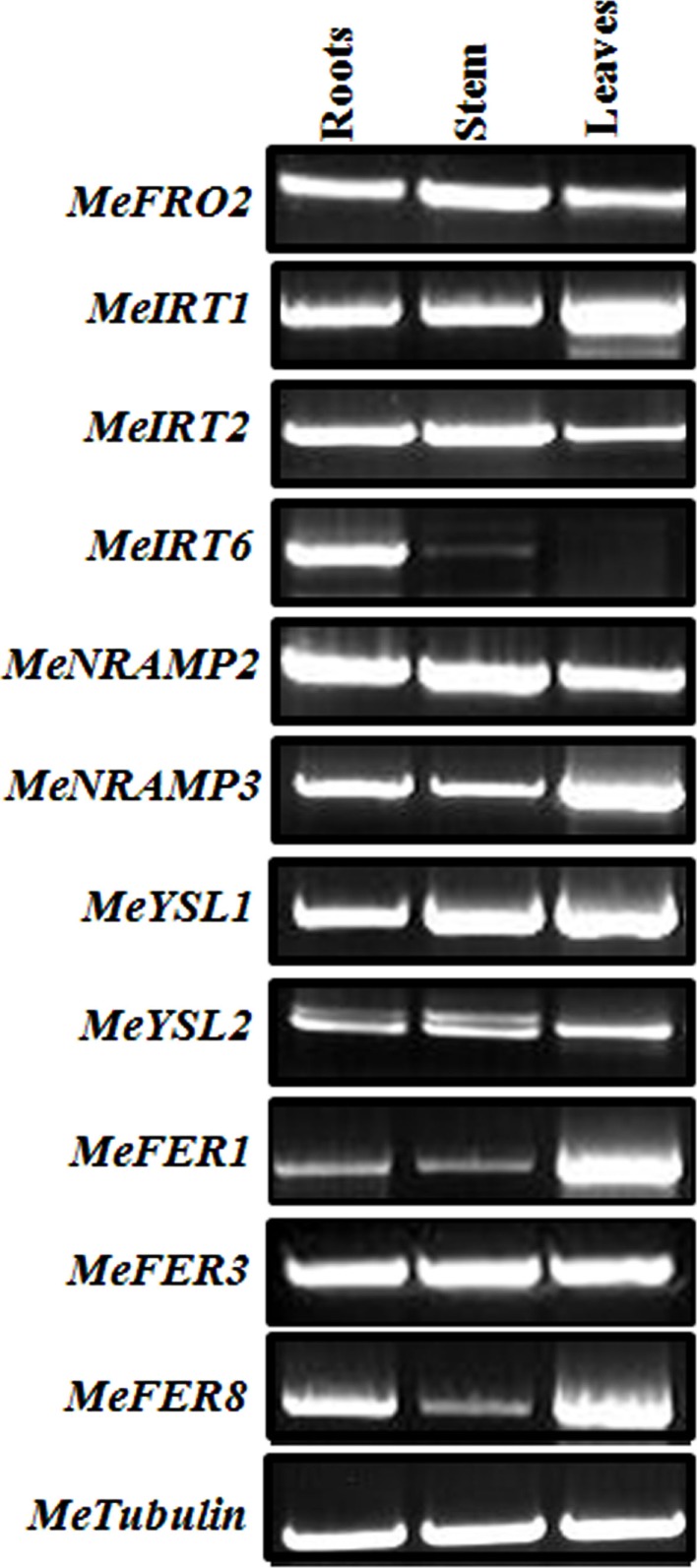
**Differential expression of genes involved in iron homeostatis in wild-type cassava**. Semi-quantitative RT-PCR analysis of metal regulated transcript abundance and expression of select metal homeostasis genes in wild-type cassava tissues. Tissues (roots, stem, and leaves) were collected at 1.5-month-old *in vitro* stage. PCR reactions were carried out with gene-specific primers as indicated in Table 1. Tubulin was used as an internal control.

**Figure 9:**
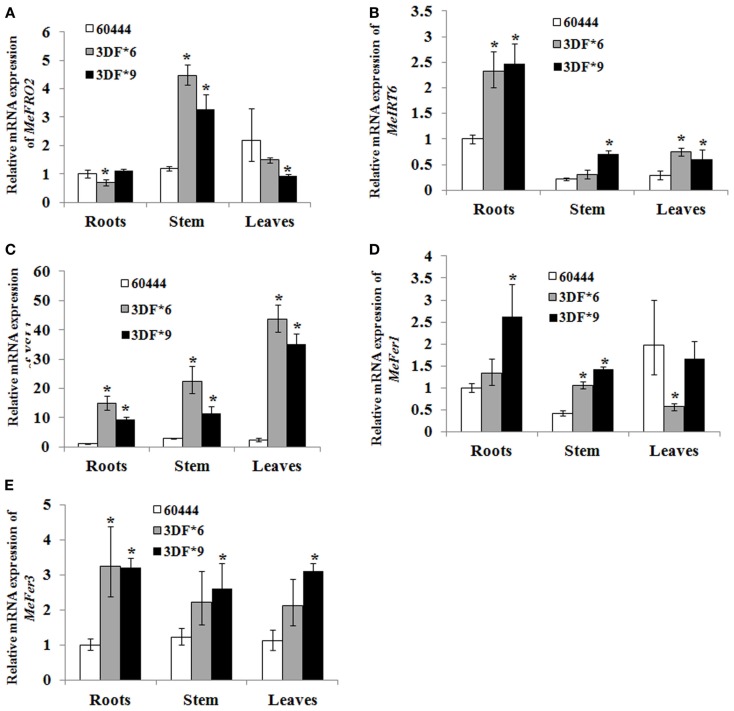
**Differential expression of metal regulated genes in *FEA1* transgenic cassava**. Relative expression (Q-RT-PCR) of metal regulated genes [**(A)**; MeFro2, **(B)**; MeIrt6, **(C)**; MeYsl1, **(D)**; MeFer1, **(E)**; MeFer3] as indicated in wild-type and two independent *FEA1* transgenic lines. Tissues (roots, stem, and leaves) were collected at 1.5-month-old *in vitro* stage plants. Expression of the metal regulated genes was normalized to alpha tubulin. Wild-type root expression levels were adjusted to a value of 1 and all other expression values were normalized relative to this tissue. For each sample, reactions were set up in quadruplicates using two biological replicates. Error bars represent SE. The asterisk (*) indicates statistically significant differences between wild-type and transgenics, determined by Student’s *t*-test, with *P* < 0.05.

To determine whether *FEA1* expression impacted other genes involve in regulating iron homeostasis (Tables 1 and 2), we determined the relative expression of the 11 genes we identified that were presumably involved in iron homeostasis in roots, stems and leaves of wild-type and *FEA1* transgenic cassava by semi-quantitative PCR. Most of the metal-regulated genes were expressed in roots, stem, and leaves of wild-type TMS 60444 cassava line (Figure 8). *MeIRT6*, however, was not detected in stem and leaves of wild-type plants using semi-quantitative PCR (Figure 8). Out of the 11 genes, *MeFRO2*, *MeIRT6*, *MeYSL1*, *MeFER1*, and *MeFER3* were selected for quantitative PCR analysis in both wild-type and *FEA1* transgenic lines based on the fact that complete gene sequences were available for these genes. Analyses of gene expression patterns in various tissues of wild-type and transgenic plants indicated significant differences in the expression patterns of the targeted genes. In transgenic cassava expressing the *FEA1* gene (3DF*9), *MeIRT6* was detectable only in root tissues in both wild-type and transgenic lines and could not be detected in stem and leaf tissues suggesting that *IRT* protein plays a major role in transporting iron from soil into the roots. *MeYSL1*, *MeFER1*, and *MeFER3* were abundantly expressed in all the tissues of wild-type and transgenic lines (data not shown) suggesting that the YSL protein plays a key role in the long distance transport of iron within the plant. Enhanced expression of ferritin in transgenic roots was consistent with the increased root storage levels of iron in these plants. Real-time PCR results suggested that *MeFRO2* was expressed in all the tissues of control plants. It is important to note that there was significant decrease in *MeFRO2* expression in the roots of the highest iron accumulating *FEA1* transgenic plants (3DF*6) when compared with the control plants (Figure 9A) suggesting that *FEA1* expressing plants are iron sufficient. Consistent with the semi-quantitative PCR results, *MeIRT6* is found to be highly expressed in roots when compared with the other tissues such as stem and leaf in both wild-type and FEA1 transgenic lines (Figure 9B). Finally, *MeYSL1*, *MeFER2*, *MeFER6* expression was upregulated in *FEA1* transgenic plants when compared to wild type suggesting these gene products may play a major role in root to shoot iron translocation and an overall role in iron homeostasis in cassava (Figures 9C–E).

## Discussion

In this study, we report the transformation and root-specific expression of the *Chlamydomonas* *FEA1* codon-optimized gene in cassava. We observed over 100-fold changes in root iron concentrations in wild-type and FEA1 transgenic plants as they transitioned from fibrous to storage roots. At 9 and 12 months of age, however, the iron concentrations in storage roots were greater than at 6 months age, perhaps representing a slower relative accumulation of dry matter compared to iron accumulation. Significantly, at harvest age (12 months), mature storage roots expressing the *FEA1* gene had threefold higher iron levels (36 ppm) than wild-type roots. Significantly, these iron levels are sufficient to meet the RDA of 18 mg Fe in a 500 g meal (White and Broadley, 2005). While we have not directly determined the bioavailability of the additional iron in transgenic plants, it has been demonstrated that the iron present in wild-type cassava is readily available in human tissue culture Caco-II studies (Ariza-Nieto et al., 2006; Lung’aho and Glahn, 2009). Furthermore, the enhanced expression of ferritin in FEA1 transgenic roots would suggest that the additional Fe is stored as ferritin complexes which are very bioavailable.

Previous attempts to biofortify crops with iron have utilized a variety of strategies. Wirth et al. (2009) reported a sixfold increase in Fe levels (1–6 ppm) in polished rice expressing three genes including; a *Phaseolus vulgaris ferritin* gene, an *Aspergillus fumigates phytase* gene, and an *Arabidopsis thaliana NAS1* (nicotianamine synthase) gene. These genes are involved in Fe storage, bioavailability, and transport, respectively. Expression of phytase would presumably make iron more bioavailable from rice since phytate complexes iron making it largely unavailable for uptake in the gut. Ferritin is a plastidial iron storage protein whose overexpression would presumably increase iron accumulation as well as bioavailabilty (Wirth et al., 2009). NAS catalyzes the synthesis of nicotianamine which is involved in iron chelation and uptake from soil as well as transport in plant vasculature tissue (Wirth et al., 2009). The increase in iron levels observed in rice grains from transgenic plants expressing all three genes was limited to a narrow cell layer within the endosperm. Since this tissue represents only a small fraction of the rice grain biomass, total iron accumulation in the grain was thus limited. Polished rice grains from wild-type and transgenic plants had an iron concentration of 1 and 6 ppm, respectively. Iron levels in whole brown rice of transgenic plants increased only 30% from 10 to 13 ppm. At these iron concentrations nearly 1.4 kg of brown rice or 3.6 kg of polished rice would need to be consumed to meet the RDA for iron, assuming all of the iron is bioavailable and none is lost during food preparation. Additional genetic approaches have also resulted in similar increases in iron levels in rice. Cheng et al. (2007) reported a twofold increase in the iron content in polished rice grains as a result of point mutation in the *naat1* gene. The *naat1* gene product is involved in the biosynthesis of deoxymugineic acid which is responsible for the chelation and absorption of Fe (III) from soil. Maximum iron levels observed in rice expressing the mutant form of *naat1* were identical (6 ppm) to those observed in transgenic lines co-expressing *ferritin*, *phytase*, and *NAS*. Interestingly, rice plants expressing the mutant *naat1* gene hyper accumulate cadmium when it is present in the root environment (Cheng et al., 2007). These results demonstrate the need for metal selective approaches for crop iron biofortification.

Targeted expression of ferric chelate reductases (FRO) has also been used to enhance (biofortify) plant iron content (Robinson et al., 1999; Connolly et al., 2003; Vasconcelos et al., 2006). FRO reduces Fe (III) to Fe (II) thus increasing its solubility and is the rate limiting step in iron uptake (Yi and Guerinot, 1996; Ghandilyan et al., 2006). Constitutive overexpression of a leaf iron-chelate reductase from *Arabidopsis* (AtFRO6) in tobacco driven by the CaMV 35S promoter was shown to result in a twofold increase in ferric reductase activity in the leaf but an insignificant increase in enzyme activity in other plant organs (Li et al., 2011). Concomitant with this increase in ferric reductase activity there was a twofold increase in iron and chlorophyll content in the leaves of transgenic plants, and reduced the susceptibility to iron deficiency chlorosis (Li et al., 2011).

Expression of the iron assimilatory protein FEA1, a microalgal periplasmic protein that presumably functions as a ferrous iron chaperonin (Rubinelli et al., 2002; Merchant et al., 2006; Allen et al., 2007). Emerging studies suggest, however, that the FEA1 protein integrates into the membrane and complements both iron transporter and endosymbiosis mutants of yeast in an energy-dependent manner, consistent with its possible function as a membrane transporter (manuscript in preparation). This is also the first time a non-plant gene involved in iron assimilation has been shown to increase iron accumulation in a crop plant. These results, as well as previous observations of enhanced iron accumulation in transgenic yeast and Arabidopsis expressing the *FEA1* gene suggest that the *FEA1* gene functions in the absence of obligate interactions with other proteins with the exception of ferric reductase since the FEA1 protein transports only ferrous iron (Narayanan et al., 2011). This is noteworthy since many iron uptake systems have low metal specificity and are capable of moving a variety of toxic divalent heavy metals as well as iron. For example, the iron transporter *irt1* is known to transport cadmium, although mutant forms of *irt1* have high iron specificity (Eide et al., 1996; Fox and Guerinot, 1998). Consistent with the observation that expression of the *FEA1* gene in yeast does not enhance uptake of competing metals, we observed that expression of the *FEA1* gene in cassava had no affect on root or leaf zinc levels. In contrast, expression of ferritin and nicotiamine synthase as well as the mutated form of the *naat1* gene all resulted in increased zinc levels in plants (Cheng et al., 2007; Wirth et al., 2009).

Importantly, iron levels were not altered in leaves of transgenic cassava plants expressing the *FEA1* gene in storage roots. Consistent with the normal distribution of leaf iron levels in transgenic plants, plant morphology, and growth rates were found to be normal. These results suggest that transgenic plants were able to maintain metal homeostasis between leaves and roots even when a strong iron sink was generated in storage roots by expression of the *FEA1* gene with the storage-tissue, root-specific, patatin promoter. It is not unexpected that leaves of cassava have higher Fe concentrations than roots given the iron demands in leaves for photosynthesis, and other metabolic pathways (Palmer and Guerinot, 2009). It has been demonstrated in other plants that about 90% of the iron that is taken up by roots is transported to leaves from which iron is redistributed to other parts of the plant (Colangelo and Guerinot, 2006; Kim and Guerinot, 2007; Palmer and Guerinot, 2009). Since leaf iron levels in transgenic and wild-type cassava plants were comparable, it was hypothesized that other plant iron homeostasis genes and gene products were able to compensate for the increased storage root sink strength in FEA1 transgenic plants.

To test this hypothesis, we assessed the relative expression levels of a variety of genes encoding proteins involved in metal transport and accumulation. We observed that the *MeIRT6*, *MeYSL1*, *MeFER2*, and *MeFER6* genes were all up regulated in *FEA1* transgenic plants when compared to wild type (Figure 9). Interestingly, *MeFRO2* was found to be down regulated in roots of FEA1 transgenic plants that accumulated the highest iron levels but elevated in stems and leaves. In agreement with this observation, transgenic plants also had reduced levels of ferric reductase enzyme activity compared to wild-type fibrous roots (Figure 6). These results suggest that *FEA1* expressing roots more efficiently take up ferrous iron than wild-type plants reducing the need for elevated ferric reductase activity. Interestingly, *MeIRT6*, iron transporter gene expression levels were elevated twofold in roots of transgenic plants but not in stems or leaves. Since *FEA1* was expressed in root storage tissues but not in root epidermal tissues, it is not unexpected that increasing root Fe sink strength in storage roots requires greater expression of *MeIRT6*, presumably to meet the increased Fe demands. It is well established that YSL iron-chelate transporters appear to be involved in movement of iron in the vasculature (DiDonato et al., 2004; Chu et al., 2010). The elevated expression of *MeYSL1* in transgenic cassava is consistent with the trends observed in other tissues reflecting the increased storage root sink strength for Fe. Similarly, ferritin gene expression is elevated (threefold) in transgenic plants, particularly in the storage roots where *FEA1* is expressed (Ravet et al., 2009). It is well established that ferritin genes have a distinct tissue-specific expression in plants. In *Arabidopsis*, *AtFER1*, *AtFER4*, and *AtFER3* are expressed in vegetative organs but not in seeds where AtFER2 is preferentially expressed (Petit et al., 2001). In our study, *MeFER3* was expressed to a greater level in transgenic plants relative to wild-type than was *MeFER1* suggesting that *MeFER3* plays an important role in root Fe storage (Figure 9E). Up regulation of *MeYSL* and ferritin expression in cassava suggests that these gene products also play a major role in root-shoot translocation and in iron homeostasis in cassava. These altered patterns of gene expression in transgenic plants expressing the *FEA1* gene under control, of the patatin promoter suggest that additional iron accumulation in roots may be achieved by; (1) elevated root hair-specific expression of ferric reductase and *IRT1* (increased source strength), and (2) elevated expression of ferritin in storage root tissues (increased sink strength).

In conclusion, we have demonstrated that overexpression of a codon-optimized *FEA1* gene from the unicellular alga, *Chlamydomonas*, in cassava roots storage tissues results in increased iron accumulation. These results demonstrate that transgenic approaches can be an effective strategy to biofortify staple crops that cannot be substantially improved by conventional breeding approaches or without impacting the levels of other metals in the plant. Reflecting the complex regulation of iron sink-source relationships, it appears that increasing the Fe sink strength in cassava storage roots results in altered patterns of expression of iron transporter and storage genes that compensate for the threefold increase in root iron content allowing leaves and other organs to have sufficient iron.

## Author Contributions

Conceived and designed the experiments: Richard T. Sayre, Uzoma E. Ihemere, and Narayanan N. Narayanan. Construction of FEA1 vector, cassava transformation, and molecular analysis of FEA1 transgenic plants: Uzoma E. Ihemere. Bioinformatics and differential expression of metal regulated genes: Narayanan N. Narayanan. Analyzed the data: Richard T. Sayre, Uzoma E. Ihemere, and Narayanan N. Narayanan. Contributed reagents/materials/analysis tools: Richard T. Sayre, Narayanan N. Narayanan, and Uzoma E. Ihemere. Wrote the paper: Richard T. Sayre, Uzoma E. Ihemere, and Narayanan N. Narayanan.

## Conflict of Interest Statement

The authors declare that the research was conducted in the absence of any commercial or financial relationships that could be construed as a potential conflict of interest.

## Footnotes

^1^http://gcua.schoedl.de/

^2^http://titan.biotec.uiuc.edu/cassava/

^3^http://www.phytozome.net/cassava

^4^http://frodo.wi.mit.edu/cgi-bin/primer3/primer3_www.cgi

## Appendix

**Table A1:** **Forward primers for the codon-optimization of *Chlamydomonas**FEA1* gene for expression in cassava**.

Orientation	FEA1 Forward primers
F1	GCACAGTTAAC**CCCGGG**ATGTCTGTCGGATTTCTGGTCCTC
F2	GCTCTGGGCGCTCTTGTCGTGGCTACTGCTCAGCCAACTACT
F3	ACTGGCACTAGATTCGAGGGTTTCTCTTACGCTGGCAATGTC
F4	ATTGGCTATGTGAACATGACTATGGACTACTGCGACATCAAG
F5	GCCGCCATGGCTGCTGGCAACTTCACCGAGGCTCTGTCCATC
F6	TACTCTACTGGCAAGAACTCTTTCTCTGGCCTGGCTAGAAGA
F7	ACCTTCTTCAGATTCGCCTCTTACATCACCGCCAACGGCTCC
F8	GTGGAGCCACTGCACGACTCCATCCTGGCCGGCAAGGACACT
F9	TCCTCCCTGGACGCCGCCATCAGGGCTGCCCTGGCCGACGGC
F10	AAGGCCACCCTGGCCGCCGGTCTGATCCAGGTGGGCACTCTC
F11	AAGTACCACCTGCACGAGGTGGATGAGGCCTACAACAAGATC
F12	AAGACCTACCTGGCTGACGGCACCGGCAACCTCACCAACCTG
F13	GTTTCTGACGCCTCTGGCGCACCACACAACGTGGATGAGGCC
F14	TGGGCTCTGTGGGCCGGCGGCGCCGCCAACAACTGCGGCACC
F15	CTGTCCGGCTGGGCCTCCTCCCTGGGCGCCGCCATGGGCACC
F16	ACCTTCCTCGGCAAGAGCTATGTCAACACCGCCATGATCAAC
F17	ACCGTCAATGAGATGCTGGCCGCCGCCAGACTGTCTACCCTG
F18	AACATCCAAGCCTACGACGCCGCTAGAACTAACGAGGTCAGA
F19	CTGCTGACCCTGCTGGGCCTGCAGGGCGTGTCCGTGGCCGCT
F20	TACACCGCTGACGCCGCCGCCGCCTGCAAGAGACCAGCCGCC
F21	GAGGTGGAGGATGCCAAGACCATGATCGCCGTGCACTGGGCT
F22	TACCTGGAGCCTATGCTCAAGCTGAGAAACTTCAAGGCCTCC
F23	GCCGTGACCGAGCTGCACCACCAGCTCACCGCCTCCAAGCTG
F24	AGCTACAAGAAGGTGGCCGCCGCTGTGAAGGGCGTGCTGTCT
F25	GCTATGGGCAGAAGATCCAGCGAGCTGGGTGCCCCACAGTCC
F26	GCCATCATTGCCGCCAACTGGAAGTGCAGCTCCAAGACCCTG
F27	AGAAGCATTGCTTAATTGGGAACATGATGGCATTGTGCTGGA
F28	AGATTGGGACTTGACACTGACTAGTTAGATAGGGCAACTCAG
F29	GCAATAGGAATCGTTAGGACCTTTAGGGAAGCTGTCATGAGC
F30	ACTGTTTGCGTGTACATATCAGTTGTGGATGGAGAAGGTGGG
F31	TAATGCCACGTTGCCGCCTTCCAGTGACTGCAAAGAAGCTAT
F32	GCTTGATTGCTTTGAGGAAGGACTCAATGCTCATCAATGAGG
F33	GTCATGATGAAATTAAATGGCCAATGTGGGGGTAAGGTTCTT
F34	TCTATGACCCCAGGTTGAAGGGTTGTGTTTGTTTGCGGCACT
F35	TGAGGTCAGTGGTAAAGACACCCAGTGCCTGCCGCTTCAGCC
F36	TGTGATCAGCTCATATGCACAAAGGCTGGAGGAGAGGAGCTG

**Table A2:** **Reverse primers for codon-optimization of *Chlamydomonas FEA1* gene for expression in cassava**.

Orientation	FEA1 reverse primer 5′–3′
R1	CATGGA**GAGCTC**ACAGTATTACATTACAGCTCCTCTCCTCCA
R2	GCCTTTGTGCATATGAGCTGATCACAGGCTGAAGCGGCAGGC
R3	ACTGGGTGTCTTTACCACTGACCTCAAGTGCCGCAAACAAAC
R4	ACAACCCTTCAACCTGGGGTCATAGAAAGAACCTTACCCCCA
R5	CATTGGCCATTTAATTTCATCATGACCCTCATTGATGAGCATT
R6	GAGTCCTTCCTCAAAGCAATCAAGCATAGCTTCTTTGCAGTC
R7	ACTGGAAGGCGGCAACGTGGCATTACCCACCTTCTCCATCCA
R8	CAACTGATATGTACACGCAAACAGTGCTCATGACAGCTTCCC
R9	TAAAGGTCCTAACGATTCCTATTGCCTGAGTTGCCCTATCTA
R10	ACTAGTCAGTGTCAAGTCCCAATCTTCCAGCACAATGCCATC
R11	ATGTTCCCAATTAAGCAATGCTTCTCAGGGTCTTGGAGCTGC
R12	ACTTCCAGTTGGCGGCAATGATGGCGGACTGTGGGGCACCCA
R13	GCTCGCTGGATCTTCTGCCCATAGCAGACAGCACGCCCTTCA
R14	CAGCGGCGGCCACCTTCTTGTAGCTCAGCTTGGAGGCGGTGA
R15	GCTGGTGGTGCAGCTCGGTCACGGCGGAGGCCTTGAAGTTTC
R16	TCAGCTTGAGCATAGGCTCCAGGTAAGCCCAGTGCACGGCGA
R17	TCATGGTCTTGGCATCCTCCACCTCGGCGGCTGGTCTCTTGC
R18	AGGCGGCGGCGGCGTCAGCGGTGTAAGCGGCCACGGACACGC
R19	CCTGCAGGCCCAGCAGGGTCAGCAGTCTGACCTCGTTAGTTC
R20	TAGCGGCGTCGTAGGCTTGGATGTTCAGGGTAGACAGTCTGG
R21	CGGCGGCCAGCATCTCATTGACGGTGTTGATCATGGCGGTGT
R22	TGACATAGCTCTTGCCGAGGAAGGTGGTGCCCATGGCGGCGC
R23	CCAGGGAGGAGGCCCAGCCGGACAGGGTGCCGCAGTTGTTGG
R24	CGGCGCCGCCGGCCCACAGAGCCCAGGCCTCATCCACGTTGT
R25	GTGGTGCGCCAGAGGCGTCAGAAACCAGGTTGGTGAGGTTGC
R26	CGGTGCCGTCAGCCAGGTAGGTCTTGATCTTGTTGTAGGCCT
R27	CATCCACCTCGTGCAGGTGGTACTTGAGAGTGCCCACCTGGA
R28	TCAGACCGGCGGCCAGGGTGGCCTTGCCGTCGGCCAGGGCAG
R29	CCCTGATGGCGGCGTCCAGGGAGGAAGTGTCCTTGCCGGCCA
R30	GGATGGAGTCGTGCAGTGGCTCCACGGAGCCGTTGGCGGTGA
R31	TGTAAGAGGCGAATCTGAAGAAGGTTCTTCTAGCCAGGCCAG
R32	AGAAAGAGTTCTTGCCAGTAGAGTAGATGGACAGAGCCTCGG
R33	TGAAGTTGCCAGCAGCCATGGCGGCCTTGATGTCGCAGTAGT
R34	CCATAGTCATGTTCACATAGCCAATGACATTGCCAGCGTAAG
R35	AGAAACCCTCGAATCTAGTGCCAGTAGTAGTTGGCTGAGCAG
R36	TAGCCACGACAAGAGCGCCCAGAGCGAGGACCAGAAATCCGA

## References

[B1] Allen M. D. del Campo J. A. Kropat J. Merchant S. S. (2007). *FEA1*, *FEA2*, and *FRE1*, encoding two homologous secreted proteins and a candidate ferrireductase, are expressed coordinately with *FOX1* and *FTR1* in iron-deficient *Chlamydomonas reinhardtii*. Eukaryotic Cell 6, 1841–1852 10.1128/EC.00205-07 17660359 PMC2043389

[B2] Arias-Garzon D. I. (1997). Genetic Engineering Approaches to Improve Agronomic Traits in Cassava (Manihot esculenta Crantz). Ph.D. thesis, The Ohio State University, Columbus

[B3] Ariza-Nieto M. Sanchez M. T. Heller L. I. Hu Y. Welch R. M. Glahn R. P. (2006). Cassava (*Manihot esculenta*) has high potential for iron biofortification. FASEB J. 20, A624

[B4] Bereczky Z. Wang H. Y. Schubert V. Ganal M. Bauer P. (2003). Differential regulation of *nramp* and *irt* metal transporter genes in wild type and iron uptake mutants of tomato. J. Biol. Chem. 278, 24697–24704 10.1074/jbc.M301365200 12709425

[B5] Bevan M. (1984). Binary *Agrobacterium* vectors for plant transformation. Nucleic Acid Res. 12, 8711–8721 10.1093/nar/12.22.8711 6095209 PMC320409

[B6] Bohn L. Meyer A. S. Rasmussen S. K. (2008). Phytate: impact on environment and human nutrition. A challenge for molecular breeding. J. Zhejiang Univ. Sci. B 9, 165–191 10.1631/jzus.B0710640 18357620 PMC2266880

[B7] Bouis H. E. (2003). Micronutrient fortification of plants through plant breeding: can it improve nutrition in man at low cost? Proc. Nutr. Soc. 62, 403–411 10.1079/PNS2003262 14506888

[B8] Bruggemann W. Mass-Kantel K. Moog P. R. (1993). Iron uptake in leaf mesophyll cells: the role of plasma membrane bound ferric chelate reductases. Planta 190, 151–155 10.1007/BF00196606

[B9] Charles A. L. Sriroth K. Huang T.-C. (2005). Proximate composition, mineral contents, hydrogen cyanide and phytic acid of 5 cassava genotypes. Food Chem. 92, 615–620 10.1016/j.foodchem.2004.08.024

[B10] Chavez A. L. Bedoya J. M. Iglesias C. Ceballos H. Roca W. (2000). Iron, carotene, and ascorbic acid in cassava roots and leaves. Food Nutr. Bull. 21, 410–413

[B11] Cheng L. Wang F. Shou H. Huang F. Zheng L. He F. Li J. Zhao F.-J. Ueno D. Ma J. F. Wu W. (2007). Mutation in nicotianamine aminotransferase stimulated the Fe(II) acquisition system and led to iron accumulation in rice. Plant Physiol. 145, 1647–1657 10.1104/pp.107.107912 17951455 PMC2151683

[B12] Chenna R. Sugawara H. Koike T. Lopez R. Gibson T. J. Higgins D. G. Thompson J. D. (2003). Multiple sequence alignment with the Clustal series of programs. Nucleic Acids Res. 31, 3497–3500 10.1093/nar/gkg500 12824352 PMC168907

[B13] Chu H. H. Chiecko J. Punshon T. Lanzirotti A. Lahner B. Salt D. E. Walker E. L. (2010). Successful reproduction requires the function of *Arabidopsis* yellow Stripe-Like1 and Yellow Stripelike3 metal–nicotianamine transporters in both vegetative and reproductive structures. Plant Physiol. 154, 197–210 10.1104/pp.110.159103 20625001 PMC2938154

[B14] Colangelo E. P. Guerinot M. L. (2006). Put the metal to the petal: metal uptake and transport throughout plants. Curr. Opin. Plant Biol. 9, 322–330 10.1016/j.pbi.2006.03.015 16616607

[B15] Connolly E. L. Campbell N. H. Grotz N. Prichard C. L. Guerinot M. L. (2003). Overexpression of the FRO2 ferric chelate reductase confers tolerance to growth on low iron and uncovers posttranscriptional control. Plant Physiol. 133, 1102–1110 10.1104/pp.103.025122 14526117 PMC281606

[B16] Connolly E. L. Guerinot M. (2002). Iron stress in plants. Genome Biol. 3, 1024–1030 10.1186/gb-2002-3-8-reviews1024 PMC139400 12186653

[B17] Curie C. Panaviene Z. Loulergue C. Dellaporta S. L. Briat J. F. Walker E. L. (2001). Maize yellow stripe1 encodes a membrane protein directly involved in Fe(III) uptake. Nature 409, 346–349 10.1038/35053080 11201743

[B18] DiDonato R. J. Jr. Roberts L. A. Sanderson T. Eisley R. B. Walker E. L. (2004). *Arabidopsis* Yellow Stripe-Like2 (YSL2): a metal-regulated gene encoding a plasma membrane transporter of nicotianamine–metal complexes. Plant J. 39, 403–414 10.1111/j.1365-313X.2004.02128.x 15255869

[B19] Eide D. Broderius M. Fett J. Guerinot M. L. (1996). A novel iron-regulated metal transporter from plants identified by functional expression in yeast. Proc. Natl. Acad. Sci. U.S.A. 93, 5624–5628 10.1073/pnas.93.20.10735 8643627 PMC39298

[B20] Fox T. C. Guerinot M. L. (1998). Molecular biology of cation transport in plants. Annu. Rev. Plant Physiol. Plant Mol. Biol. 49, 669–696 10.1146/annurev.arplant.49.1.669 15012250

[B21] Frossard E. Bucher M. Machler F. Mozafar A. Hurrell R. (2000). Potential for increasing the content and bioavailability of Fe, Zn, and Ca in plants for human nutrition. J. Sci. Food Agric. 80, 861–879 10.1002/(SICI)1097-0010(20000515)80:7<861::AID-JSFA601>3.0.CO;2-P

[B22] Gamborg O. L. Miller R. A. Ojima K. (1968). Nutrient requirements of suspension cultures of soybean root tissue. Exp. Cell Res. 50, 151–158 10.1016/0014-4827(68)90403-5 5650857

[B23] Ghandilyan A. Vreugdenhil D. Aarts M. G. M. (2006). Progress in the genetic understanding of plant iron and zinc nutrition. Physiol. Plant. 126, 407–417 10.1111/j.1399-3054.2006.00646.x

[B24] Goto F. Yoshihara T. Shigemoto N. Toki S. Takaiwa F. (1999). Iron fortification of rice seed by the soybean ferritin gene. Nat. Biotechnol. 17, 282–286 10.1038/7029 10096297

[B25] Guerinot M. L. (2000). The ZIP family of metal transporters. Biochim. Biophys. Acta 1465, 190–198 10.1016/S0005-2736(00)00138-3 10748254

[B26] Henriques R. Jásik J. Klein M. Martinoia E. Feller U. Schell J. Pais M. S. Koncz C. (2002). Knock-out of *Arabidopsis* metal transporter gene *IRT1* results in iron deficiency accompanied by cell differentiation defects. Plant Mol. Biol. 50, 587–597 10.1023/A:1019942200164 12374293

[B27] Ihemere U. E. (2003). Somatic Embryogenesis and Transformation of Cassava for Enhanced Starch Production. Ph.D. thesis, The Ohio State University, Columbus, OH

[B28] Ihemere U. Arias-Garzon D. Lawrence S. Sayre T. (2006). Genetic modification of cassava for enhanced starch production. Plant Biotechnol. J. 4, 453–465 10.1111/j.1467-7652.2006.00195.x 17177810

[B29] Ihemere U. E. Siritunga D. Sayre R. T. (2008). “Cassava,” in Compendium of Transgenic Crop Plants, Vol. 7, eds Kole C. Hall T. C. (Hoboken: Wiley-Blackwell), 177–198

[B30] Ishimaru Y. Suzuki M. Tsukamoto T. Suzuki K. Nakazono M. Kobayashi T. Wada Y. Watanabe S. Matsuhashi S. Takahashi M. Nakanishi H. Mori S. Nishizawa N. K. (2006). Rice plants take up iron as an Fe^3+^-phytosiderophore and as Fe^2+^. Plant J. 45, 335–346 10.1111/j.1365-313X.2005.02624.x 16412081

[B31] Jeong J. Guerinot M. L. (2009). Homing in on iron homeostasis in plants. Trends Plant Sci. 14, 280–285 10.1016/j.tplants.2009.02.006 19375375

[B32] Kim S. A. Guerinot M. L. (2007). Mining iron: iron uptake and transport in plants. FEBS Lett. 581, 2273–2280 10.1016/j.febslet.2007.05.053 17485078

[B33] Leyva-Guerrero E. Narayanan N. Ihemere U. Sayre R. T. (2012). Iron and protein biofortification of cassava: lessons learned. Curr. Opin. Biotechnol. 23, 257–264 10.1016/j.copbio.2011.12.009 22226461

[B34] Li L.-Y. Cai Q.-Y. Yu D.-S. Guo C.-H. (2011). Overexpression of *AtFRO6* in transgenic tobacco enhances ferric chelate reductase activity in leaves and increases tolerance to iron-deficiency chlorosis. Mol. Biol. Rep. 38, 3605–3613 10.1007/s11033-010-0472-9 21104018

[B35] Livak K. J. Schmittgen T. D. (2001). Analysis of relative gene expression data using realtime quantitative PCR and the 2ΔΔCt method. Methods 25, 402–408 10.1006/meth.2001.1262 11846609

[B36] Lucca P. Hurrell R. Potrykus I. (2001). Genetic engineering approaches to improve the bioavailability and the level of iron in rice grains. Theor. Appl. Genet. 102, 392–397 10.1007/s001220051659

[B37] Lucca P. Hurrell R. Potrykus I. (2002). Fighting iron deficiency anemia with iron-rich rice. J. Am. Coll. Nutr. 21, 184S–190S 12071303 10.1080/07315724.2002.10719264

[B38] Lung’aho M. G. Glahn R. P. (2009). *In vitro* estimates of iron bioavailability in some Kenyan complementary foods. Food Nutr. Bull. 30, 145–152 19689093 10.1177/156482650903000206

[B39] Marfo E. K. Simpson B. K. Idowu J. S. Oke O. L. (1990). Effect of local food processing on phytate levels in cassava, cocoyam, yam, maize, sorghum, rice, cowpea, and soybean. J. Agric. Food Chem. 38, 1580–1585 10.1021/jf00097a032

[B40] Maser P. Thomine S. Schroeder J. I. Ward J. M. Hirschi K. Sze H. Talke I. N. Amtmann A. Maathuis F. J. M. Sanders D. Harper J. F. Tchieu J. Gribskov M. Persans M. W. Salt D. E. Kim S. A. Guerinot M. L. (2001). Phylogenetic relationships within cation transporter families of *Arabidopsis*. Plant Physiol. 126, 1646–1667 10.1104/pp.126.4.1646 11500563 PMC117164

[B41] Masuda H. Suzuki M. Morikawa K. Kobayashi T. H. N. Takahashi M. Saigusa M. Mori S. Nishizawa N. (2008). Increase in iron and zinc concentrations in rice grains via the introduction of barley genes involved in phytosiderophore synthesis. Rice 1, 100–108 10.1007/s12284-008-9007-6

[B42] Mathews H. Schopke C. Carcamo R. Chavarriaga P. Fauquet L. Beachy R. N. (1993). Improvement of somatic embryogenesis and plant recovery in cassava. Plant Cell Rep. 12, 328–333 10.1007/BF00237429 24197258

[B43] Mayer J. E. Pfeiffer W. H. Beyer P. (2008). Biofortified crops to alleviate micronutrient malnutrition. Curr. Opin. Plant Biol. 11, 166–170 10.1016/j.pbi.2008.01.007 18314378

[B44] Merchant S. S. Allen M. D. Kropart J. Moseley J. L. Long J. C. Tottey S. Terauchi A. M. (2006). Between a rock and a hard place: trace element nutrition in *Chlamydomonas*. Biochim. Biophys. Acta 1763, 578–594 10.1016/j.bbamcr.2006.04.007 16766055

[B45] Msikita W. Ihemere U. E. Siritunga D. Sayre R. T. (2006). “Cassava (*Manihot esculenta* Crantz),” in Agrobacterium Protocols: Methods in Molecular Biology Book Series, 344, Vol. 2, ed. Wang K. (Totowa, NJ: Humana Press), 13–24 10.1385/1-59745-131-2:13 17033047

[B46] Murakawa H. Bland C. E. Willis W. T. Dallman P. R. (1987). Iron deficiency andneutrophil function: different rates of correction of the depressions in oxidative burst and myeloperoxidase activity after iron treatment. Blood 695, 1464–1468 3032307

[B47] Murashige T. Skoog F. (1962). A revised medium for rapid growth and bioassays with tobacco tissue cultures. Physiol. Plant. 15, 473–497 10.1111/j.1399-3054.1962.tb08052.x

[B48] Murphy J. F. Riordan J. Newcombe R. G. Coles E. C. Pearson J. F. (1986). Relation of haemoglobin levels in first and second trimesters to outcome of pregnancy. Lancet 1, 992–995 10.1016/S0140-6736(86)91269-9 2871331

[B49] Narayanan N. N. Ihemere U. Chiu W. T. Siritunga D. Rajamani S. Singh S. Ode S. Sayre R. T. (2011). The iron assimilatory protein, FEA1, from *Chlamydomonas reinhardtii* facilitates iron-specific metal uptake in yeast and plants. Front. Plant Sci. 2:67 10.3389/fpls.2011.00067 22639604 PMC3355668

[B50] Nestel P. Bouis H. E. Meenakshi J. V. Pfeiffer W. (2006). Biofortification of staple food crops. J. Nutr. 136, 1064–1067 16549478 10.1093/jn/136.4.1064

[B51] Nguema A. (2011). Economic Benefits of Meeting Nutritional needs Through Biofortified Cassava: An Example from Nigeria and Kenya. MS thesis.

[B52] Nicholas K. B. Nicholas H. B. J. (1997). Genedoc: A Tool for Editing and Annotating Multiple Sequence Alignments. Distributed by the author. Available at: http://www.psc.edu/biomed/genedoc

[B53] le Qu Q. Yoshihara T. Ooyama A. Goto F. Takaiwa F. (2005). Iron accumulation does not parallel the high expression level of ferritin in transgenic rice seeds. Planta 222, 225–233 10.1007/s00425-005-1530-8 15821927

[B54] Palmer C. M. Guerinot M. L. (2009). Facing the challenges of Cu, Fe and Zn homeostasis in plants. Nat. Chem. Biol. 5, 333–340 10.1038/nchembio.166 19377460 PMC3085079

[B55] Petit J. M. Briat J. F. Lobreaux S. (2001). Structure and differential expression of the four members of the *Arabidopsis thaliana* ferritin gene family. Biochem. J. 359, 575–582 10.1042/0264-6021:3590575 11672431 PMC1222178

[B56] Ravet K. Touraine B. Boucherez J. Briat J. Gaymard F. Cellier F. (2009). Ferritins control interaction between iron homeostasis and oxidative stress in *Arabidopsis*. Plant J. 57, 400–412 10.1111/j.1365-313X.2008.03698.x 18826427

[B57] Robinson N. J. Groom S. J. Groom Q. J. (1997). The *froh* gene family from *Arabidopsis thaliana*: putative iron-chelate reductases. Plant Soil 196, 245–248 10.1023/A:1004258225806

[B58] Robinson N. J. Procter C. M. Connolly E. L. Guerinot M. L. (1999). A ferric-chelate reductase for iron uptake from soils. Nature 397, 694–697 10.1038/17800 10067892

[B59] Rosahl S. Schmit R. Schell J. Willmitzer L. (1986). Isolation and characterization of a gene from *Solanum tuberosum* encoding patatin, the major storage protein of potato tubers. Mol. Gen. Genet. 203, 214–220 10.1007/BF00333957

[B60] Rubinelli P. Siripornadulsil S. Gao-Rubinelli F. Sayre R. T. (2002). Cadmium- and ironstress-inducible gene expression in the green alga *Chlamydomonas reinhardtii*: evidence for H43 protein function in iron assimilation. Planta 215, 1–13 10.1007/s00425-001-0711-3 12012236

[B61] Saitou N. Nei M. (1987). The neighbor-joining method: a new method for reconstructing phylogenetic trees. Mol. Biol. Evol. 4, 406–425 3447015 10.1093/oxfordjournals.molbev.a040454

[B62] Sautter C. Poletti S. Zhang P. Gruissem W. (2006). Biofortification of essential nutritional compounds and trace elements in rice and cassava. Proc. Nutr. Soc. 65, 153–159 10.1079/PNS2006488 16672076

[B63] Savoie N. Rioux F. M. (2002). Impact of maternal anemia on the infant’s iron status at 9 months of age. Can. J. Public Health 93, 203–207 12050988 10.1007/BF03405001 PMC6980000

[B64] Sayre R. T. Beeching J. Cahoon E. Egesi C. Fauquet C. Fellman J. Fregene M. Gruissem W. Mallowa S. Manary M. Maziya-Dixon B. Mbanaso A. Schachtman D. P. Siritunga D. Taylor N. Vanderschuren H. Zhang P. (2011). The BioCassava plus program: biofortification of cassava for sub-saharan Africa. Annu. Rev. Plant Biol. 62, 251–272 10.1146/annurev-arplant-042110-103751 21526968

[B65] Siritunga D. Sayre R. T. (2003). Generation of cyanogen-free transgenic cassava. Planta 217, 367–373 10.1007/s00425-003-1005-8 14520563

[B66] Siritunga D. Sayre R. T. (2004). Engineering cyanogen synthesis and turnover in cassava (*Manihot esculenta*). Plant Mol. Biol. 56, 661–669 10.1007/s11103-004-3415-9 15630626

[B67] Stephenson L. S. Latham M. C. Ottesen E. A. (2000). Global malnutrition. Parasitology 121, S5–S22 10.1017/S0031182000006491 11386691

[B68] Tamura K. Dudley J. Nei M. Kumar S. (2007). MEGA4: molecular evolutionary genetics analysis (MEGA) software version 4.0. Mol. Biol. Evol. 24, 1596–1599 10.1093/molbev/msm092 17488738

[B69] Vasconcelos M. Datta K. Oliva N. Khalekuzzaman M. Torrizo L. Krishnan S. Oliveira M. Goto F. Datta S. K. (2003). Enhanced iron and zinc accumulation in transgenic rice with the ferritin gene. Plant Sci. 164, 371–378 10.1016/S0168-9452(02)00421-1

[B70] Vasconcelos M. Eckert H. Arahana V. Graef G. Grusak M. A. Clemente T. (2006). Molecular and phenotypic characterization of transgenic soybean expressing the *Arabidopsis* ferric chelate reductase gene, *FRO2*. Planta 224, 1116–1128 10.1007/s00425-006-0293-1 16741749

[B71] Vert G. Grotz N. Dédaldéchamp F. Gaymard F. Guerinot M. L. Briat J.-F. Curie C. (2002). IRT1, an Arabidopsis transporter essential for iron uptake from the soil and for plant growth. Plant Cell 14, 1223–1233 10.1105/tpc.001388 12084823 PMC150776

[B72] White P. J. Broadley M. R. (2005). Biofortifying crops with essential mineral elements. Trends Plant Sci. 10, 586–593 10.1016/j.tplants.2005.10.001 16271501

[B73] Wirth J. Poletti S. Aeschlimann B. Yakandawala1 N. Drosse1 B. Osorio S. Tohge T. Fernie A. R. Gunther D. Gruissem W. Sautter C. (2009). Rice endosperm iron biofortification by targeted and synergistic action of nicotianamine synthase and ferritin. Plant Biotechnol. J. 7, 631–644 10.1111/j.1467-7652.2009.00430.x 19702755

[B74] Yi Y. Guerinot M. L. (1996). Genetic evidence that induction of root Fe (III) chelate reductase activity is necessary for iron uptake under iron deficiency. Plant J. 10, 835–844 10.1046/j.1365-313X.1996.10050835.x 8953245

[B75] Zimmermann M. Hurrell R. F. (2007). Nutritional iron deficiency. Lancet 370, 511–520 10.1016/S0140-6736(07)61235-5 17693180

