# Erratum: Iron biofortification and homeostasis in transgenic cassava roots expressing an algal iron assimilatory protein, FEA1

**DOI:** 10.3389/fpls.2013.00492

**Published:** 2013-12-03

**Authors:** Richard Sayre

**Affiliations:** New Mexico Consortium at Los Alamos National Labs, Biolabs, Los AlamosNM, USA

**Keywords:** cassava, iron, transgenes, FEA1, biofortification

In this article, there is a typo error and needs to be changed and corrected.

Pg. 12; Line 22 - MeFER2 and MeFER6 should be replaced by “MeFER1 and MeFER3.”

